# Chemical Profile and Safety Assessment of a Food-Grade Acetogenin-Enriched Antimicrobial Extract from Avocado Seed

**DOI:** 10.3390/molecules24132354

**Published:** 2019-06-26

**Authors:** Dariana G. Rodríguez-Sánchez, Adriana Pacheco, Raúl Villarreal-Lara, Martín R. Ramos-González, Perla A. Ramos-Parra, Sergio Granados-Principal, Rocío I. Díaz de la Garza, Gerardo García-Rivas, Carmen Hernández-Brenes

**Affiliations:** 1 Tecnologico de Monterrey, Escuela de Ingeniería y Ciencias, Ave. Eugenio Garza Sada 2501, Monterrey, NL 64849, Mexico; 2 Tecnologico de Monterrey, Centro de Biotecnologia-FEMSA, Ave. Eugenio Garza Sada 2501, Monterrey, NL 64849, Mexico; 3 Tecnologico de Monterrey, Escuela de Medicina y Ciencias de la Salud, Ave. Eugenio Garza Sada 2501, Monterrey, NL 64849, Mexico; 4 Tecnologico de Monterrey, Medicina Cardiovascular y Metabolómica. Batallón de San Patricio, 112 Col. Real de San Agustín, San Pedro Garza García, NL 66278, Mexico; 5 UGC de Oncología Médica, Hospital Universitario de Jaén, Avenida del Ejército Español 10, 23007 Jaén, Spain; 6 GENYO. Centre for Genomics and Oncological Research, Pfizer/University of Granada/Andalusian Regional Government, PTS Granada-Avenida de la Ilustración 114, 18016 Granada, Spain

**Keywords:** Acetogenins, avocado, safety, natural food additives, antimicrobial

## Abstract

Acetogenins are bioactive fatty acid derivatives found in avocado tissues. Their efficacy as antimicrobials has been documented and initiated interest to use them as replacements of synthetic food additives. The present work focused on evaluation of multiple analytical methodologies for detection and quantification of organic solids present in a food-grade acetogenin-enriched extract (Avosafe^®^), and on its safety evaluations using bacterial reverse mutation (AMES) tests and acute oral toxicity to rat assays. Results confirmed chemical structures of two acetogenins as present in Avosafe^®^ (AcO-avocadyne-(**0**) and AcO-avocadiene B-(**3**)), and together with seven other previously known compounds, quantified 94.74 ± 5.77% *w*/*w* of its solids as acetogenins. Safety evaluations indicated that Avosafe^®^ was non-mutagenic and had an acute median lethal oral dose (LD_50_) to rats higher than the maximum concentration tested (>2000 mg·kg^−1^), with no signs of macroscopic abnormalities in organs. Mean body weight and hematological and biochemical parameters were normal after 14 days of a single oral dose of 2000 mg·kg^−1^. The results advance scientific information on the safety of avocado seed acetogenins and also generate new knowledge on profiles and concentrations of individual acetogenins found in avocado tissues (seed, pulp, and leaves) and in Avosafe^®^.

## 1. Introduction

Lauraceous acetogenins are a family of biologically active derivatives from long chain fatty acids [1]. This family of phytochemicals has been reported to be exclusively present in species of the genus *Persea* (family: Lauraceae) [1] and are found in different varieties and tissues (peel, pulp, and seed [2], as well as in leaves [3]) of avocado fruit (*Persea americana* Mill.). Of special interest to the food industry is their bioactivity as antimicrobial additives due to their ability to inhibit germination of bacterial endospores [4,5] and to act as listericidal additives [6,7]. Initial studies on their ability to inhibit bacterial endospores were performed using acetogenin-enriched extracts purified in a laboratory setting using bioassay-guided isolation [4]. Further works then focused on the chemical characterization of an acetogenin-enriched food additive from avocado seed (78.50 ± 4.87% acetogenins), called Avosafe^®^, and the documentation of its antimicrobial activities [5,6,7]. Studies on the chemical characterization of the acetogenin-enriched extract confirmed the chemical structures of seven acetogenins as present in the enriched avocado seed extract and 21.50 ± 1.87% *w*/*w* of solids remained to be characterized [6]. Prior studies also documented that acetogenins were stable under relevant food processing conditions (temperature, pressure, salt, pH), and were capable of controlling *C. sporogenes* endospore germination [5] and *Listeria monocytogenes* outgrowth [6,7] in both laboratory culture media and within food systems that contained meat.

Based on the existing scientific evidence on antimicrobial efficacy, avocado acetogenins are regarded as promising natural alternatives for the substitution of synthetic food additives in-line with the growing trend of clean food labels [8]. This consumer trend is linked to a generalized, but questionable, belief that natural compounds, because of their occurrence in nature, are less toxic to humans than the synthetic additives that we are currently consuming [9]. However, the development of novel natural food additives requires detailed information of the chemical composition of all organic solids present in the natural extracts and unquestionably of their safety.

Knowledge on the safety of purified avocado acetogenins is scarce in the literature, since most studies have focused on studying their bioactivities. A few studies have suggested that their function in the avocado plant is to provide defense, by exerting toxicity, against potential attackers, such as chewing herbivores (i.e., insects) [1,10,11,12] and the fungal pathogen *Colletotrichum gloeosporioides* [13]. The latter effects were attributed to Persin (compound **7** in Table S1) [12,13]. However, evidence related to acetogenin toxicity against non-target organisms (i.e., mammalians) is contradictory. A summary of *in vivo* biological studies that were conducted with purified acetogenins from avocado leaves or pulp is included in the supplementary information (Table S1).

The objectives of the present work were to evaluate multiple analytical methodologies for the detection and quantification of uncharacterized organic solids present in a food-grade acetogenin-enriched extract (Avosafe^®^). The new strategy combined various detection methods for identity assignment (HPLC-PDA/ELSD, HPLC-ESI-TOF-MS, and ESI-MS/MS) and was also used to improve scientific knowledge on acetogenin profiles and concentrations found in avocado tissues (seed, pulp, and leaf) of the commercial ‘Hass’ cultivar. The study also aimed to perform safety evaluations of the acetogenins present in Avosafe^®^ using bacterial reverse mutation (AMES) tests and acute oral toxicity to rat assays.

## 2. Results

### 2.1. Confirmation of Chemical Structure of Two Acetogenin Compounds Present in An Acetogenin-Enriched Avocado Seed Extract (Avosafe^®^)

**Figure 1:**
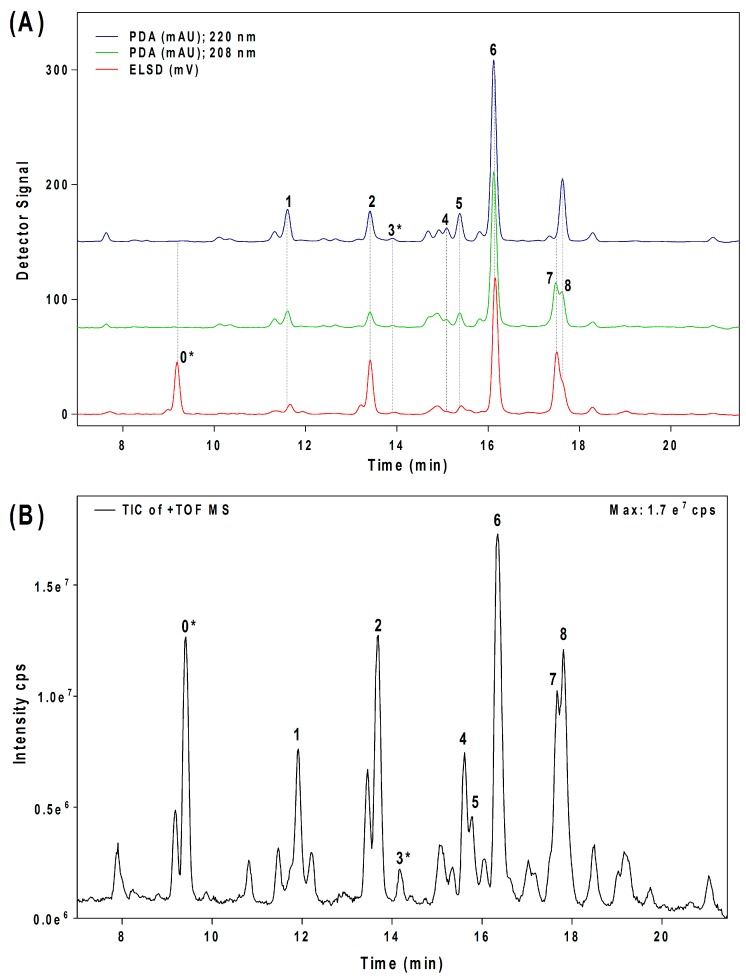
Chromatographic profiles of acetogenin molecules present in a food-grade acetogenin-enriched extract from avocado seed (Avosafe^®^). Panel (**A**) shows chromatograms obtained by HPLC-PDA/ELSD. Panel (**B**) shows chromatograms obtained by HPLC-ESI-TOF-MS. Each numbered peak corresponds to one acetogenin with numbers assigned according to elution order from HPLC column. Table 1 contains complementary information on the chemical identity of each chromatographic peak. An asterisk indicates the compounds with chemical structures assigned in this work.

Seven major chromatographic peaks (**1** to **6** and **7** in Figure 1A) were detected in the acetogenin-enriched extract (Avosafe^®^) using HPLC separation coupled to a photodiode array (PDA) detector at 220 nm. To resolve and quantify peaks **7** and **8**, detection was also conducted at 208 nm, since peak **7** had no UV absorption at 220 nm. In this work, an Evaporative Light Scattering Detector (ELSD) was also coupled to a previously reported chromatographic method [6] in order to visualize other compounds with poor UV absorption. ELSD was able to detect a new chromatographic peak that eluted earlier (**0**, Figure 1A), which was also visible using ESI-TOF-MS detection (**0**, Figure 1B) under conditions previously optimized for avocado acetogenins [4], but was not visible by PDA detection (at 208 or 220 nm). Therefore, with the use of PDA and ELS detectors in tandem, we were able to establish that Avosafe^®^ contained nine major chromatographic peaks (Figure 1A,B). The chemical structures of seven peaks corresponded to those established in prior works [2,6], which were AcO-avocadenyne (**1**), AcO-avocadene (**2**), Persediene (**4**), Persenone C (**5**), Persenone A (**6**), Persin (**7**), and Persenone B (**8**) (Table 1). However, the structures of peaks **0** and **3** remained unknown and generated further experimentation that will be described in the following paragraphs.

Further analysis of peak **0** and **3** LC-ESI-MS spectra (Figure S1A,B, respectively) confirmed that both contained a molecular ion at *m*/*z* 327 ([M + H]^+^), its corresponding sodium adduct at *m*/*z* 349 ([M + Na]^+^), and a dimer with sodium adduct at *m*/*z* 675 ([2M + Na]^+^), suggesting that they might be isomers. Moreover, both compounds displayed the characteristic ion pattern of acetogenins [15,18], showing progressive losses of water and/or acetate, producing fragments at *m*/*z* 309 ([M − H_2_O]^+^), 291 ([M − 2H_2_O]^+^), 249 ([M + H − H_2_O − CH_3_COOH]^+^), and 267 ([M + H − CH_3_COOH]^+^), which corresponded to losses of 18, 36, 78, or 60 amu from molecular ion at *m*/*z* 327 ([M + H]^+^), respectively (Figure S1A,B).

**Figure 2:**
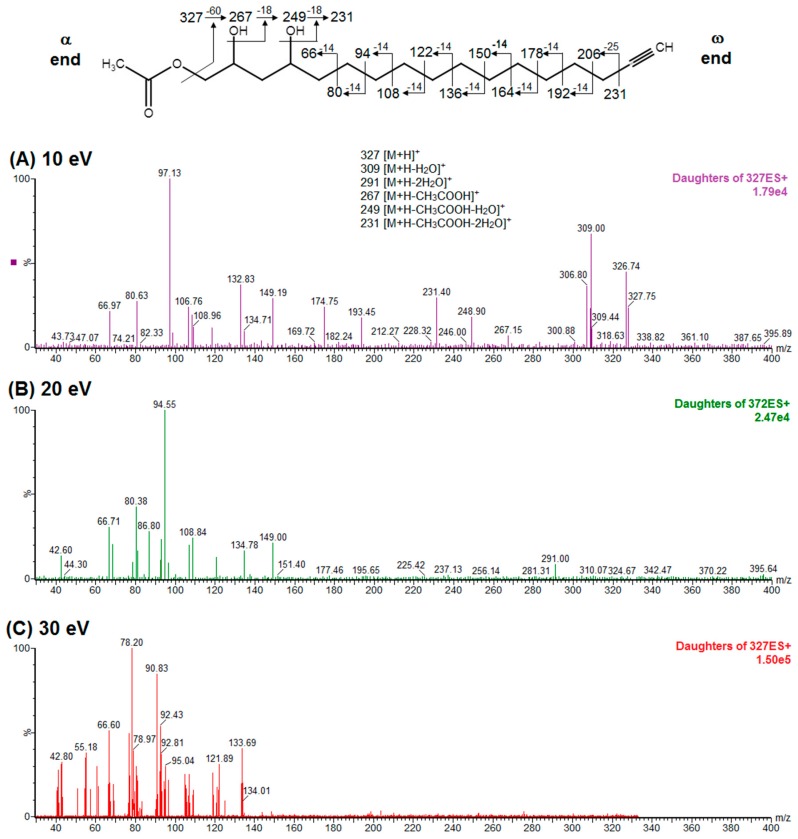
ESI-MS/MS spectra of AcO-avocadyne (labeled as compound **0** in Table 1 and Figure 1) obtained by collision-induced dissociation (CID) of the precursor ion at *m*/*z* 327 and the daughter ion at *m*/*z* 249. Data shown in panels are for collision energies of (**A**) 10 eV, (**B**) 20 eV, and (**C**) 30 eV. Note: “α end” refers to the charged side of the molecule that corresponded to oxygenated functional groups, whereas “ω end” refers to the methyl end.

To elucidate the identity of compounds **0** or **3**, they were both purified from Avosafe^®^ by preparative HPLC-PDA protocols (to a purity > 97%) [4]. Subsequently, purified compounds **0** and **3** were independently infused into the ESI source of a triple quadrupole mass spectrometer in positive-ion mode to determine their low-energy (<30 eV), collision-induced dissociation (CID) spectra (ESI-MS/MS). Information was collected on the dissociation of the precursor ion (total ion at *m*/*z* 327) after the losses of acetate and water (daughter [M + H − CH_3_COOH − H_2_O]^+^ = *m*/*z* 249). As shown in Figure 2A, CID spectra of compound **0** produced a rich pattern of peaks separated by 14 amu (from *m*/*z* 66 to 192), indicative of consecutive losses of methylene groups (CH_2_), which has been reported to be typical for the fragmentation of saturated alkyl side chains of annonaceous acetogenins [18], avofurans [19], and fatty acids [20]. From this pattern, it was possible to determine that to produce fragments with the described mass, after losing the oxygenated groups (acetoxy and hydroxyls), the compound was also losing a fragment of 25 units, which corresponded to a terminal triple bond (acetylenic, CH≡C-). Therefore, based on our results, the identity of compound **0** was assigned as 1-Acetoxy-2,4-dihydroxy-heptadec-16-yne (AcO-avocadyne (**0**), Table 1 and Figure 2), which featured a terminal triple bond (C16−C17) and an aliphatic chain of C11 (from C-5 to C-15).

**Figure 3:**
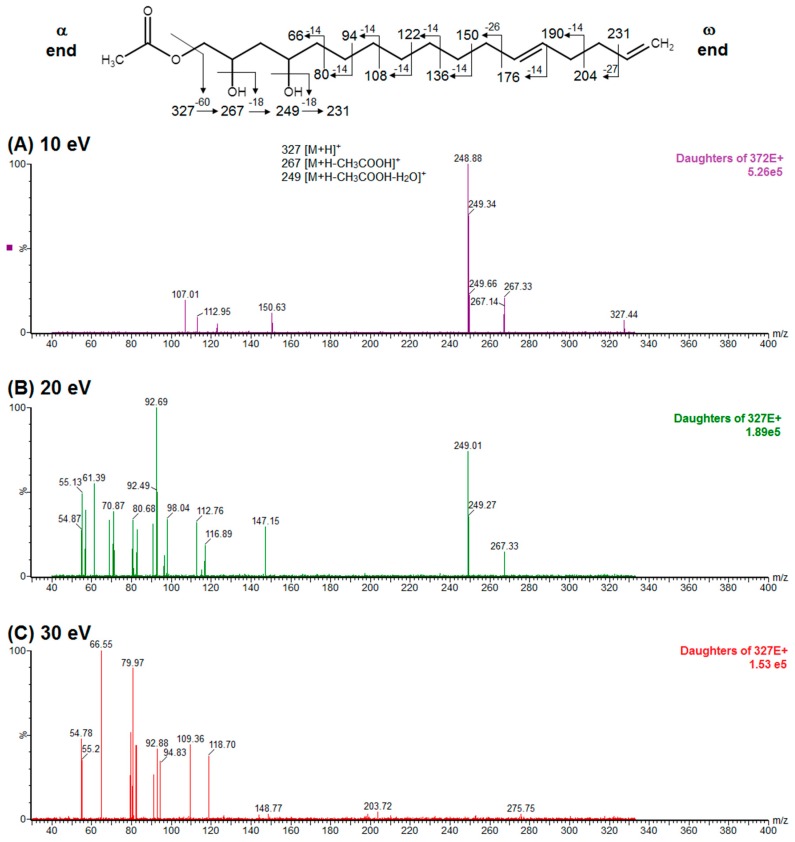
ESI-MS/MS spectra of AcO-avocadiene B (labeled as compound **3** in Table 1 and Figure 1) obtained by collision-induced dissociation (CID) of the precursor ion at *m*/*z* 327 and daughter ion at *m*/*z* 249. Data shown in panels are for collision energies of (**A**) 10 eV, (**B**) 20 eV, and (**C**) 30 eV. Note: “α end” refers to the charged side of the molecule that corresponded to oxygenated functional groups, whereas “ω end” refers to the methyl end.

Regarding the identity of compound **3**, through CID fragmentation it was possible to observe ions at *m/z* 267, 249, and 231 that corresponded to losses of acetate and two hydroxyls groups, respectively (Figure 3A,B), followed by the loss of a 27 amu portion, representative of a terminal double bond (vynilic, CH_2_=CH-) [21], which produced a fragment of *m*/*z* 204 (Figure 3C). In addition, it was possible to observe a fragment of *m*/*z* 150 when lower collision energy was applied (Figure 3A), indicating the presence of a C12−C13 double bond. A series of ions from *m*/*z* 66 to 122, each separated by 14 mass units (indicative of a saturated alkyl side chain [18,19]), were clearly visible at higher collision energies (Figure 3C). The molecular ion and fragmentation pattern observed suggested that the structure of compound **3** corresponded to 1-Acetoxy-2,4-dihydroxy-heptadeca-12,16-diene, which features a terminal double bond (C16−C17) and a C12−C13 double bond, and was named here as AcO-avocadiene B (**3**) (Table 1 and Figure 3).

To support the two previous chemical identities, CID was also conducted using an analytical standard (AcO-avocadene (**2**)), previously characterized by NMR [4]. AcO-avocadene and AcO-avocadyne (**0**) feature a similar structure that only differs by the presence of a vinylic or an acetylenic terminal bond, respectively (Table 1). As observed in Figure 2 and supplementary Figure S2, low-energy CID spectra of AcO-avocadyne (**0**) and AcO-avocadene (**2**) presented the same fragment ions at lower masses (*m*/*z* < 195). Fragmentation patterns suggested that after the loss of the oxygenated groups of these two molecules, their following site of cleavage was their corresponding terminal vynilic or acetylenic bond, producing the same subsequent fragmentation pattern. As expected, CID fragmentation of compound AcO-avocadiene B (**3**), shown in Figure 3, presented a dissimilar fragmentation pattern, since the position of double bonds within its structure was different.

HPLC-ESI-TOF-MS analyses of Avosafe^®^ also revealed presence of other minor peaks (Figure 1B). As shown in supplementary Table S2, eleven peaks presented the characteristic ion pattern reported for acetogenins [4,15]. Putative structures for the eleven peaks were included in supplementary Table S2, and were tentatively assigned based on correspondence of their ESI-TOF-MS spectra with those of other acetogenins previously reported in literature [14,16,22,23,24,25].

### 2.2. Quantification of Acetogenins Found in Avosafe^®^, a Food-Grade Acetogenin-Enriched Extract

**Figure 4:**
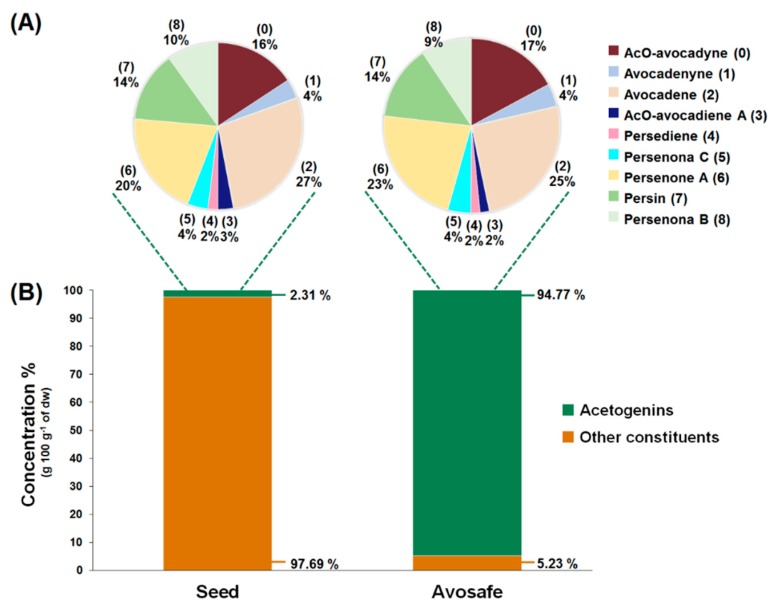
Acetogenin profiles and concentrations (dry weight basis, dw) found in avocado seed (*Persea americana* ‘Hass’) and in Avosafe^®^, a food-grade acetogenin-enriched extract from avocado seed. (**A**) Shown in pie charts are the chemical profile and relative concentrations of nine acetogenins quantified in avocado seed and Avosafe^®^. (**B**) shown in bar charts are the percent contribution of total acetogenin concentrations to the total organic solid composition of avocado seed and of Avosafe^®^. Values represent mean ± SD (*n* = 3).

Concentrations of the nine acetogenins detected in Avosafe^®^ by HPLC-PDA/ELSD are shown in Figure 4A,B. New analytical strategy indicated that the nine major compounds present in the food-grade acetogenin-enriched extract (Avosafe^®^) accounted for 94.74 ± 5.77% of its organic solids. Acetogenins with the highest percent contribution to the composition of Avosafe^®^ included AcO-avocadene (**2**), Persenone A (**6**), and AcO-Avocadyne (**0**), which represented 24.00 ± 1.84%, 21.26 ± 1.40%, and 16.24 ± 0.27% *w*/*w*, respectively. Interestingly, Avosafe^®^ exhibited a very similar acetogenin profile to avocado seed (Figure 4A), from which it is extracted. However, the food-grade extract Avosafe^®^ contained an acetogenin concentration 41-fold higher than the original levels found in seed (Figure 4A,B).

Accurate quantification of the eleven minor peaks detected in Avosafe^®^ (supplementary Table S2) remains to be achieved due to the lack of analytical standards; however, the individual contribution of each one of the eleven peaks was estimated to be between 0.34 and 1.41% (quantified at 220 nm as Persenone A (**6**) equivalents). Although quantification (in equivalents) assumed a similar spectroscopic response for all eleven peaks to that of Persenone A (**6**), results indicated that the eleven minor peaks summed approximately 8.66 ± 0.68% *w*/*w* of acetogenins as the remaining unknown solids present in Avosafe^®^. However, considering that the quantification was performed as Persenone A (**6**) equivalents, the estimation was close but not precise, since our proper quantification of the known nine peaks (Figure 4B, conducted with purified analytical standards) indicated that only 5.23% *w*/*w* of the solids remained to be characterized.

### 2.3. Individual Profiles and Quantification of Acetogenins Present in Avocado (‘Hass’ cultivar) Seed, Pulp, and Leaves using the Improved Analytical Methodology

**Figure 5:**
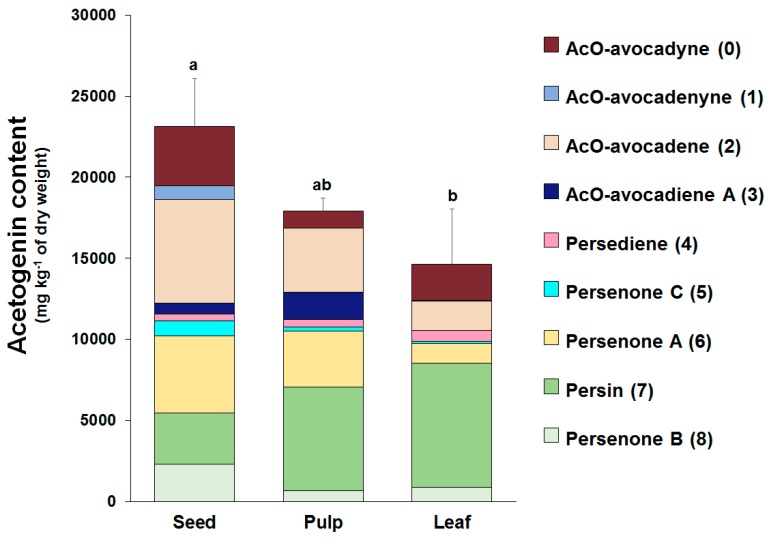
Individual and total acetogenin concentrations (dry weight basis) in seed, pulp, and leaf tissues of avocado (*Persea americana* ‘Hass’*)*. Values represent means of the corresponding biological replicates (*n* = 3–5) and error bars represent the standard deviation of the total acetogenin concentrations. Different letters indicate significant differences for the total acetogenin concentrations (Tukey HSD test, α < 0.05).

The three avocado tissues (seed, pulp, and leaves) accumulated acetogenins differently (Figure 5). The highest acetogenin contents were observed for the seed (2.31 ± 0.29% *w*/*w*, dw), followed closely by pulp (1.79 ± 0.07% *w*/*w*, dw), and then by leaf tissue (1.46 ± 0.34% *w*/*w*, dw). Profiles were also very different among tissues, for instance the main acetogenins found in the seeds included, in decreasing order of concentration, AcO-avocadene (**2**), Persenone A (**6**), and AcO-avocadyne (**0**) with relative levels of 27, 20, and 16% (*w*/*w*), respectively. In contrast, Persin (**7**) was found to be the most abundant acetogenin in leaf and pulp, accounting for 52 and 36% (*w*/*w*) of total acetogenins present in each tissue, respectively, while it only represented 14% of total acetogenins present in the seed. Other remarkable differences among tissues were that AcO-avocadenyne (**1**) was only present in seed, while AcO-avocadiene B (**3**) was not detected in leaf.

### 2.4. Bacterial Reverse Mutation Test (AMES Test)

In order to comply with the regulatory guidelines of the bacterial reverse mutation tests [26], the highest concentrations tested were 5000 µg Avosafe ^®^ plate^−1^ (corresponding to 4738.5 µg of total acetogenins plate^−1^). As indicated in the methodology section, the in vitro experiments on the mutagenic potential of Avosafe^®^ were evaluated using histidine dependent auxotrophic mutants of *Salmonella typhimurium* (strains TA1535, TA1537, TA98, and TA100) and a tryptophan-dependent mutant of *Escherichia coli*, strain WP2 uvrA (pKM101).

No significant increases (*p < 0.05*) in the number of revertant colonies of tested strains were observed either at the level of exposure of 5000 μg Avosafe^®^ plate^−1^ (Table 2) or at lower concentrations (supplementary Table S3). Results were compared to the negative controls with and without exogenous metabolic activation system (S9 mix). In the AMES test methodology, 2- to 3-fold increases relative to vehicle controls are not considered biologically relevant [27], therefore, our observations for Avosafe^®^ suggested no mutagenic nor cytotoxic potential in the bacterial reverse mutation test.

### 2.5. Study I-Acute Oral Toxicity in Rats (Fixed Dose Method): Acute Median Lethal Oral Dose (LD_50_) and Macroscopic Pathology Analyses

As shown in Table S1, previous studies suggested that acetogenins (mainly Persin (**7**)) caused toxicological signs in lactating mice, at doses ranging from 60–100 mg·kg^−1^ of bw [28], corresponding to 30–50 mg·kg^−1^ of bw for rat, considering interspecies data correction factors [29]. Therefore, the sighting investigation in this study started at 300 mg of Avosafe^®^ solids kg^−1^ bw, using 1 single rat. Since no negative effect was observed, a dose of 2000 mg·kg^−1^ was administered to a second rat, and the treatment also resulted in no signs of toxicity. Hence, following the standard procedure, the main study was carried out with a group of 4 rats that were administered with 2000 mg of Avosafe^®^ solids kg^−1^. After the established observation period of 14 days [30], no deaths were observed in experimental animals, either during sighting investigation or in the main study. In addition, none of the animals presented any of the clinical signs associated to toxicity (coma, prostration, hyperactivity, loss of righting reflex, ataxia, or difficult breathing [31]), whereas all of them were considered to have achieved satisfactory body weight gains throughout the study (data not shown). Together, data indicated that the acute median lethal oral dose (LD_50_) to rats of the acetogenin-enriched extract appeared to be greater than 2000 mg of Avosafe^®^ solids kg^−1^ bw (with an acetogenin content of 94.74% *w*/*w*).

Macroscopic pathology analyses indicated no abnormalities in the animals included in the study (Table S4). All macroscopic tissue examinations of the animals were normal at study termination on day 15. Tissues evaluated included brain, caecum, duodenum, heart, kidneys, small and large intestine, liver, lungs and bronchi, spleen, stomach, subcutaneous tissue, and urinary bladder.

### 2.6. Study II-Acute Oral Toxicity in Rats (Single Dose of 2000 mg·kg^−1^): Clinical Observations, Hematology, and Serum Biochemistry

#### 2.6.1. Clinical Observations and Body Weight

A second acute oral toxicity experiment was conducted based on results of the first acute oral toxicity study (with highest dose of 2000 mg of Avosafe^®^ kg^−1^ bw), in which neither of the treated rats died nor showed significative signs of toxicity after 15 days of observation. In the second study a detailed examination was performed on a group of 3 rats administered with 2000 mg of Avosafe^®^ kg^−1^ bw, in which clinical signs, hematology, serum biochemistry, and body weight were compared to a control group that received only the PG vehicle.

Treatments were administered orally using a plastic gastric catheter in a single bolo and the rats were monitored for postural, behavioral, and physiological changes every hour for the first 6 h and then on a daily basis until completion of the 14-day study length. Results from observations are summarized in Table 3 and did not exhibit any relevant changes between treated groups (PG vehicle and Avosafe^®^). All animals in the study presented a slight reduction in movement following oral administration; observations that are attributed to the stress of gastric catheter introduction. A single individual of the Avosafe^®^ group presented soft evacuation during the first 6 h of the study, possibly related with the lipidic nature of the compound, however it recovered normal feces consistency after a few hours.

**Figure 6:**
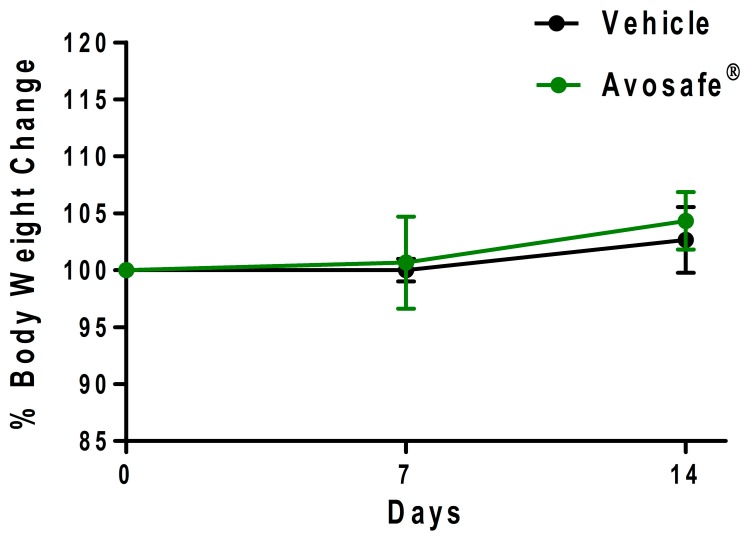
Body weight (bw) change in rats over time (14 days) following acute oral administration (2000 mg·kg^−1^) of a food-grade extract from avocado seed (Avosafe^®^), with an acetogenin purity of 94.74%. Values represent mean ± SD (*n* = 3).

The animals in the study did not show any alteration either in sleep or in the feeding patrons. To properly document observation, the body weight (bw) changes from days 0 to 7 and from days 8 to 14 were calculated for each animal group. Both groups kept gaining weight as expected with no significant differences (at *p <* 0.05) detected between them, as shown in Figure 6.

#### 2.6.2. Hematology and Serum Biochemistry Analysis

Values of hematological cells and hemoglobin-related parameters for the blood samples collected at the end of the study (day 14) are shown in Table 4. No relevant differences were observed for the treated animals and values were within the normal ranges reported for rats [32], including the hemoglobin values that showed statistical difference when compared to control values (*p* < 0.05).

A serum biochemical analysis was performed in order to confirm the absence of toxicity assessed by the main renal and hepatic molecular blood indicators, as well as by lipidic and osmotic ions concentrations in plasma. Table 5 summarizes all measured parameters. Under the experimental conditions used, it was concluded that the food-grade acetogenin enriched extract was safe for oral ingestion, since it did not alter principal organic functions when compared with the control group. A slight increase in serum glucose was observed in the Avosafe^®^ treated group, however the value was considered normal for rats and rodents [32].

#### 2.6.3. Macroscopic Observations

At the end of the study, all animals were properly euthanized as described in the methodology. The visual appearance of all organs was evaluated, and the main organs of interest were resected and weighed to compare both groups in terms of relative weight. Macroscopic examination of heart, brain, liver, and the pair of kidneys was normal. Ratios of organ weight relative to body weight are shown in Table 6, and showed no significant differences between groups. No abnormalities were observed in macroscopic appearance of the evaluated organs or in their weight (relative to body weight), therefore no histopathological examination was conducted.

## 3. Discussion

### 3.1. Identification and Quantification of Acetogenins Found in Avocado Seed and in a Food-Grade Acetogenin-Enriched Extract Obtained from Avocado Seed (Avosafe^®^)

Bioactive properties of avocado acetogenins have been studied by various authors [1,4,10,11,14,22,23,33,34,35]. However, other than our research group [2,6], only a few works have quantified these compounds in different avocado fruit tissues [13,23,28,36,37,38] or in the extracts used for bioactivity assessments [5,6]. The absence of quantitative data in the literature is possibly due to challenges for the isolation, purification, and chemical identification of acetogenin analytical standards, which are needed as quantification references. Therefore, most of the quantification efforts have focused on one acetogenin, Persin (compound **7** in Table 1), mainly because of interest in its protective role against phytopathogens and insects [3,13,36,38,39], or a result of interest in the study of its toxicology [28]. Among the methodologies that have been proposed for the quantification of acetogenins are HPLC-RI [36], HPLC-UV [13], UPLC or HPLC-PDA [2,6,39], HPLC-ELSD [23], and GC-FID [3,40], whereas qualitative evaluation and structure elucidation have been typically carried out by HPLC-MS [14,15,23,35], GC-MS [19], or direct infusion to MS detector [34], accompanied by NMR.

On the topic of avocado acetogenin characterization, our research group reported an HPLC method that coupled PDA or ESI-TOF-MS detectors for qualitative and quantitative evaluations of these molecules in different tissues and extracts [2,6]. Using the reported methodology, it was possible to identify and quantify eight acetogenins in an acetogenin-enriched avocado seed extract (Avosafe^®^) with antibacterial properties, which accounted for 78.50 ± 4.87% *w*/*w* of total organic solids present [5,6]. Quantification was conducted using purified analytical standards of confirmed chemical identity [2,4]. However, the analytical method was not able to detect compounds lacking a UV chromophore, as is the case of some lipids [41,42]. The sensitivity of LC-MS was also not adequate to assure identity and quantification, since the ionization step can be affected by instrumental, solubility, and compound-related parameters or properties [42].

In the present work we incorporated an ELSD detector to the already reported HPLC-PDA or ESI-TOF-MS methodologies [2,6]. The ELSD detector, differing from the other detectors, was only sensitive to the intensity of light scattered by the solid particles of the sample (mass of vaporized analytes) [42,43]. Interestingly, as shown in Figure 1A, the ELSD detector allowed visualization of an additional molecule (compound **0**), which was untraceable by the PDA detector. Compound **0** was also detected by our HPLC-ESI-TOF-MS established method (Figure 1B); therefore, the total amount of chromatographic peaks visualized through ELSD and ESI-TOF-MS was the same (Figure 1A,B). The area under the curve of the unknown compound **0,** as detected by ELSD and ESI-TOF-MS, was similar to that of a major seed component (AcO-avocadene (**2**)), therefore compound **0** was presumed to be present in the extract at relevant concentrations (Figure 1A,B). Considering the information, further experiments were performed to purify the compound by preparative chromatography in order to elucidate its chemical structure and for its use as the HPLC-ELSD quantification analytical standard.

LC-ESI-MS spectra of the purified compound **0** ([M + H]^+^ = *m*/*z* 327) suggested that it was an structural isomer of compound **3** (Figure S1), which was also previously reported by our group as another unknown putative acetogenin (UPA) present in avocado fruit [2,6]. Additionally, LC-ESI-MS fragmentation patterns of both compounds (Figure S1) were similar to those of other three acetogenin molecules present in avocado seed, as reported by Ramos-Jerz (2007) [14], which only differed in the location of unsaturated bonds.

On the task of giving identity to compounds **0** and **3**, we were not able to assign them to specific structures previously reported in the literature and based on their LC-ESI-MS spectra alone [14]. The main limitation was that the determination of double bond position within carbon chain of a molecule using mass spectrometry is a challenging task, since under electron impact conditions double bond migration can take place [20]. However, the task of positioning double bonds has been eased by discovery of the charge-remote fragmentation (CRF) phenomenon in mass spectrometry, which characterizes bond cleavages occurring at locations distant (remote) from the charged moiety of an organic ion subjected to collisional-induced dissociation (CID) [44]. CID spectra can be structurally informative, since they display fragment ions produced from cleavage of each carbon–carbon bond, however the presence of an unsaturation in a molecule suppresses (but does not eliminate) the cleavage of the unsaturated bond, therefore, fragments corresponding to cleavage of carbon–carbon single bonds are more abundant [45]. In this sense, CRF has been used to successfully determine chain length and locations of unsaturations in lipids, including fatty acids and other organic compounds containing alkyl chains [46,47]. Some variations of CRF include the use of high (keV) or low-energy (<100 eV) CID, analysis of intact molecules, chemically derivatized molecules, or reactions to increase sensitivity, as well as analysis in positive or negative ion modes [47].

In the present study, compounds **0** and **3** were independently infused into the ESI source of a triple quadrupole mass spectrometer to produce positive ions. Mass selection was conducted (precursor ion: M + H^+^ = *m*/*z* 327 and daughter ion: M + H − CH_3_COOH − H_2_O^+^ = *m*/*z* 249), as well as fragmentation using low-energy CID (<30 eV). As shown in Figure 2 and Figure 3, position differences of unsaturated bonds between compounds **0** and **3** resulted in different fragmentation patterns. CID spectra of compound **0** yielded fragment ions that revealed the presence of a saturated acyl chain with a terminal acetylenic bond, which corresponded to structural motifs of 1-Acetoxy-2,4-dihydroxy-heptadec-16-yne (AcO-avocadyne (**0**), Table 1 and Figure 2), which was previously reported as present in avocado seed [14] and pulp [15]. On the other hand, CID spectra of compound **3** indicated the presence of two different double bonds located at C12-C15 and C16-C17, as in 1-Acetoxy-2,4-dihydroxy-heptadeca-12,16-diene, previously described by Ramos-Jerz (2007) [14], and named here as AcO-avocadiene B (**3**) (Table 1 and Figure 3).

An additional low-energy CID experiment was also conducted for another NMR-confirmed acetogenin standard (AcO-avocadene (**2**)) to learn about the CRF phenomenon with a molecule of the same family and of known identity. AcO-avocadene (**2**) differed from AcO-avocadyne (**0**) only in the presence of a vinylic bond instead of an acetylenic bond. Data made it evident that after losing the representative ion fragment of their differential terminal bond (*m/z* at 206 and 204, respectively), the fragment ions produced for both compounds were very similar (Figure 2 and Figure S2), providing additional evidence for the identity assignment as AcO-avocadyne (**0**).

The choice of CID as the strategy to establish the location of the unsaturated bonds of acetogenins **0** and **3**, in this work, had various strengths according to prior authors. One of them was the infusion of high purity (>97%) compounds in independent runs, and another one was prior knowledge of the molecular weight of the parent ion subjected to dissociation [48]. The use of CID to provide convincing structural evidence in a prior publication [46] also strengthened confidence in our experimental design.

CID spectra observed in the present work for AcO-avocadyne (**0**) and AcO-avocadiene B (**3**) also shared common motifs with low-energy CID fragmentation patterns previously reported for unsaturated fatty acids [20], possibly due to resemblance with their long aliphatic chains. However, differences in the peculiar locations of the unsaturations present in AcO-avocadyne (**0**)**,** AcO-avocadiene B (**3**), and AcO-avocadene (**2**), such as terminal or unconjugated types, and on their bond types (triple and double), offered a valuable opportunity to compare spectral features that corresponded to each isomer (as previously discussed).

Different collision energies were used in our CID experiments, following recommendations of Gross (2000) [46], and also considering the contrasting unsaturated bonds featured by the three pure acetogenins infused. Data generated on different functional groups gave us the opportunity to analyze dissociation patterns of the different lipidic molecules (including our results and data previously generated for fatty acids [46]). To facilitate our description of CID data, in subsequent mentions referring to acetogenins or fatty acids, the side of the molecule containing the charge-site that corresponded to oxygenated functional groups (acetoxy or carboxyl, respectively) will be referred to as the “α end” and the methyl end of the molecule as the “ω end”.

A characteristic feature observed for the CRF phenomenon of unsaturated fatty acids was the presence of abundant fragments that corresponded to the cleavage of the carbon-carbon single bonds adjacent to every existing unsaturation, on its α side of the unsaturation (vinylic cleavage, C=C-) [20]. Likewise, fragment ions at *m/z* 150 and 204 in CID spectra of AcO-avocadiene B (**3**) (Figure 3A,C) reflected the occurrence of that vinylic cleavage, which confirmed the presence of two double bonds at C12−C13 and C16−C17. In contrast, for AcO-avocadyne (**0**) and AcO-avocadene (**2**), the fragment ions reflecting this type of cleavage (acetylenic C≡C-, and vinylic cleavage, respectively), which were expected at *m/z* 206 and 204, respectively, were not observed at any of the evaluated collision energies (Figure 2 and Figure S2, respectively). Remarkably, the fragment ion of highest *m/z* generated from a carbon–carbon single bond cleavage of the latter two molecules was at *m/z* 192, suggesting an allylic carbon–carbon cleavage (C=C-C-) on the α side of the unsaturation. Fragments denoting allylic cleavages (on both the α and ω side of the unsaturation) have been reported as the most abundant ions generated from unsaturated lipids subjected to high-energy CID [44], however its occurrence combined with vinylic cleavage has also been reported at low-energies [20].

Moreover, as shown in Figure 3A, at the lowest evaluated collision energy CID (10 eV,) CID spectra of AcO-avocadiene B (**3**) presented only two fragment ions representative of carbon–carbon single bond cleavage (*m*/*z* at 108 and 150), and the number of fragments and their abundance increased as the collision energy increased (>20 eV, Figure 3B,C). However, for AcO-avocadyne (**0**) and AcO-avocadene (**2**), the richest number of fragments ions were produced at the lowest collision energy (10 eV) (Figure 2 and Figure S2, respectively) and were separated by 14 amu (from *m*/*z* 66 to 192), representative of cleavage of carbon–carbon single bonds [20]. The latter observation appeared to indicate that for this set of molecules, the presence of a single terminal bond in AcO-avocadyne (**0**) and AcO-avocadene (**2**) favored the occurrence of carbon-carbon single bond cleavage at low energies (10eV), and in contrast, the presence of two double bonds on AcO-avocadiene B (**3**) somehow suppressed the occurrence of carbon–carbon single bond cleavage at low energies (10eV).

In the present work we observed qualitative and quantitative improvements in the analytical methodology as a result of coupling ELSD. From the qualitative perspective, contrary to PDA, ESLD is a chromophore-independent method of detection [49]. Therefore, once the identity of compound **0** was assigned as AcO-avocadyne (**0**), it was possible to understand that the presence of a terminal triple bond made it a poor chromophore, a characteristic trait of alkynes, with very low UV-absorption at wavelengths below 200 nm [49]. Once the purification of the AcO-avocadyne (**0**) was achieved, its quantification by means of ELSD was also possible. As shown in Figure 4A, the compound represented 17.05 ± 1.35% *w*/*w* of the total acetogenins present in Avosafe^®^ (or 16.24 ±1.64% *w*/*w* of its total organic solids). New knowledge on the identity of solids contained in Avosafe^®^ increased the concentrations of its fully characterized solids from 78.50 ± 4.87% to 94.74 ± 5.77% acetogenins *w*/*w*. Information also allowed us to establish that AcO-avocadyne (**0**) was among the three major constituents of Avosafe^®^, only after Persenone A (**6**) and AcO-avocadene (**2**) (Figure 1). An additional observation worth mentioning was that even though peak areas for AcO-avocadyne (**0**) by ELSD and ESI-TOF-MS detectors were very similar to those of AcO-avocadene (**2**) and Persin (**7**) (Figure 1A,B), once properly quantified, concentrations resulted at 32% and 21% lower and higher, respectively. The latter observation was attributed to the nonlinear responses of specific analyte concentrations to ELSD, since responses do not obey Beer’s Law, but are more influenced by particle sizes [50]. For these reasons, and in agreement with recently developed methods to quantify lipids using ELSD [43], in the present study calibration curves for AcO-avocadyne (**0**) were fitted to second order polynomial equations. Using AcO-avocadyne (**0**) analytical standards, the regression coefficients (r^2^) of second order equations were very close to 1, while when data was fitted to linear equations the r^2^ coefficients were lower than 0.98.

### 3.2. Improvements in the Quantification of Acetogenins Present in Avocado (‘Hass’ cultivar) Seed, Pulp, and Leaf

Analytical improvements introduced in this work allowed us to generate a more accurate profile and quantification of the acetogenins present in avocado seed, pulp, and leaf of the ’Hass‘ cultivar. Previous works from our research group provided information on the quantification of acetogenins in the pulp, seed, and peel of 22 different avocado cultivars [2]; another study focused on acetogenin analyses in seeds and pulps of the ‘Hass’ cultivar at different developmental stages [51]. However, the analytical methodologies used in prior studies quantified only 8 acetogenin molecules (compounds 1 to 8 shown in Table 1). Herein, with the introduction of ELSD detection method, we learned that concentrations were underestimated in prior studies, since AcO-avocadyne (**0**) was not observed with the PDA detector. As shown in Figure 5, the quantification of AcO-avocadyne (**0**) contributed 16%, 15%, and 6% (*w*/*w*) to the total acetogenin concentrations found in the seed, leaf, and pulp of ‘Hass’ avocado, respectively. Therefore, through this work we estimated that total acetogenin concentrations in the seed, pulp, and leaves were 9250.12 ± 1184.49, 4482.22 ± 191.55, and 4903.26 ± 1143.10 mg·kg^−1^ fresh weight (fw), respectively.

Valuable information also obtained from present study included acetogenin profiles of avocado leaves (‘Hass’ cultivar) and their quantification (Figure 5), which to the best of knowledge is being reported herein for the first time. Prior publications had only determined the contents of one acetogenin (Persin (**7**)) in leaves of different avocado cultivars [3,28,37,38], including ‘Hass’ variety [3]. In the present study, average Persin (**7**) concentrations in ‘Hass’ avocado leaves were found to be 2568.40 ± 487.28 mg·kg^−1^ (fw). Persin (**7**) levels for ‘Hass’ avocado leaves, previously reported by Carman and Handley (1990) [3], ranged from 3900 to 4500 mg·kg^−1^ (fw), values that were 1.5–1.7 times higher than those obtained in the present work. The same authors also quantified Persin **(7)** concentrations in the leaves of seventeen avocado cultivars, and their contents ranged between 400 to 4500 mg·kg^−1^ fw, of which the ‘Hass’ cultivar contained the highest Persin (**7**) levels.

Very few studies [13,23], aside from our group’s works [2,6,51], have quantified individual acetogenin compounds in avocado tissues other than leaves. For instance, Kobiler and others (1993) [13] quantified only two acetogenins, Persin **(7)** and AcO-avocadene (**2**), in avocado pulp (‘Hass’ cultivar) at full maturity. The authors reported an average Persin (**7**) content of 1520 ± 250 mg·kg^−1^ fw, which was in agreement with our results, since the pulp analyzed herein contained 1600 ± 68 mg of Persin (**7**) kg^−1^ fw (Figure 5). However, AcO-avocadene (**2**) contents reported by the authors were 1.5-times higher than concentrations obtained in the present study (1530 ± 90 vs. 990 ± 47 mg·kg^−1^ fw, respectively). In addition, Degenhardt and Hofmann [23] reported the concentration of eight acetogenins in fully ripe avocado pulp (‘Hass’ cultivar), including AcO-avocadyne (**0**), Persenone-A (**6**), and Persin (**7**) (70 ± 10, 230 ± 80, and 360 ± 30 mg·kg^−1^ fw, respectively), which were about 4-times lower than levels obtained in the present study (260 ± 33, 864 ± 41, and 1600 ± 68 mg·kg^−1^ fw, respectively). Although it is not well understood what factors can influence acetogenin concentrations, differences have been attributed to a wide variety of factors that can affect lipid metabolism, such as developmental stage, maturity, and the environment [51].

Other scientific works have also reported the presence of Persin (**7**) and other acetogenins in avocado pulp [24,36,40]; however, their results were expressed as standard graphs (i.e., peak height vs. sample weight in mg) in µg acetogenins idioblast·cells^−1^, or as ratios of acetogenin contents in different tissues, respectively. Consequently, it was not feasible to compare their data with our results or prior quantifications.

### 3.3. Insights on the Safety of a Food-Grade Avocado Seed Extract Enriched in Acetogenins (94.74% w/w Purity)

Results from the bacterial reverse mutation test (Table 2) suggested no mutagenic nor cytotoxic potential of Avosafe^®^ in the range of concentrations evaluated (5 to 5000 µg plate^−1^). Other than data generated herein, no information was found in the scientific literature on the safety evaluation of purified acetogenins using the AMES test. Therefore, the present work is possibly the first assessment of the mutagenic potential of a highly purified acetogenin extract (94.74% *w*/*w* purity, as indicated in Figure 4). However, other authors have studied the genotoxic activity of avocado seed ethanolic extracts by the micronucleus assay in rodents, and reported no genotoxic effects at extract concentrations of 250 mg·kg^−1^ [52]. The current work had the strength of studying a purified acetogenin extract, which is relevant in the design of studies on the safety assessment of avocado seed components. Prior studies with crude extracts are not considered adequate samples to study the safety of individual components of a particular plant matrix, and therefore were difficult to compare with our results. In the present work, purified acetogenins were not mutagenic in the AMES test (at the range of concentrations studied), however further research is always desirable, particularly because of their highly unsaturated nature that makes them susceptible to oxidation. The study of oxidation metabolites may be relevant in further studies, since it has been reported that aldehydic oxidation products of polyunsaturated fatty acids (PUFAS) (i.e., 2-hexenal) increased spontaneous mutation counts that doubled the negative control (in the TA100 AMES strain at 314 µg plate^−1^) [53]. Conversely, clinical studies indicated that supplementation of postmenopausal women with fish-oil-rich ω-3 PUFAS was not associated with greater in vivo lipid peroxidation [54].

In the present study, the oral toxicity to the rat study was conducted in a stepwise mode, as described in methodology; sighting was done using one animal of a single sex followed by a further confirmatory main study that used four animals, adding to a total of five animals [30]. Exposure doses were 5, 50, 300, or 2000 mg·kg^−1^, and although the design can exceptionally administer 5000 mg·kg^−1^ when justified by specific regulatory needs, the dose was not included in this study [30]. The experimental design used in the present work is known to provide reliable information on the hazardous properties of a test substance, it allowed classification of results according to the Globally Harmonized System (GHS) [31], and it maximized animal welfare considerations [30].

As described in the methodology section, PG was used as delivery vehicle, since acetogenins are lipid molecules that are not water-soluble. PG was considered a safe vehicle, since it has been proven not to be acutely toxic to three different species, and its oral LD_50_ values are reported to be between 19.7 and 24.9 g·kg^−1^ of bw [55]. The sighting investigation in this study started at 300 mg of Avosafe^®^ solids kg^−1^ bw using one single rat and no toxic effects were observed; this initial dose was selected based on previous studies, which reported toxicological signs at doses ranging from 60–100 mg of Persin (**7**) kg^−1^ of bw (Table S1) [28]. The rats also showed no negative effects when treated with a higher dose of 2000 mg·kg^−1^, therefore a second rat was treated, which also showed no signs of toxicity (Table 3, Table 4, Table 5 and Table 6). Hence, following the standard procedure, the main study involved the treatment of four rats with 2000 mg of Avosafe^®^ solids kg^−1^. After the established observation period of 14 days [30], no deaths were observed in the experimental animals, neither during the sighting investigation nor the main study. None of the animals presented any of the clinical signs associated with toxicity (coma, prostration, hyperactivity, loss of righting reflex, ataxia, or difficult breathing [31]), whereas all of them were considered to have achieved satisfactory body weight gains throughout the study (data not shown). Together, data indicated that the acute median lethal oral dose (LD_50_) of Avosafe^®^ solids evaluated in rats was greater than 2000 mg·kg^−1^ bw (extract contained 94.74% of acetogenins *w*/*w*). The experimental design used allowed us to place the results within the GHS of Classification and Labelling of Chemicals [31], which manages an international toxicological system with 5 categories. Compounds with the highest toxicity (LD_50_ ≤ 5 mg·kg^−1^ bw) are ranked in category 1, while category 5 groups compounds with relatively low acute toxicity (LD_50_ > 2000 and ≤ 5000 mg·kg^−1^ bw) but that under certain circumstances, may represent a hazard to especially vulnerable populations. Therefore, based on the results of the present study, Avosafe^®^ was classified in category 5.

Study II of acute toxicity was based on the results of the first acute oral toxicity study, described in the previous sections, in which animals were treated with the highest dose of 2000 mg·kg^−1^ bw of Avosafe^®^ and none of the treated rats died nor showed signs of toxicity after 15 days of observation. Three rats were used in the subsequent evaluations and were administered with 2000 mg·kg^−1^ of Avosafe^®^, and their clinical signs, hematology, serum biochemistry, and body weight were within normal levels and comparable to the control group, which ingested only the PG vehicle (Table 3, Table 4 and Table 5 and Figure 6)**.** Macroscopic examinations of the organs (heart, brain, liver, and kidneys) detected no abnormalities (supplementary Table S4). Ratios of organ weights relative to body weights also showed no significant differences from the control group (Table 6).

Our results from the three different safety studies indicated that the acetogenin-enriched extract did not demonstrate signs of toxicity. Comparison of our results with prior scientific reports proved to be a difficult task, since, to the best of knowledge, only four studies have assessed biological effects of purified acetogenins in mammals (including Persin (**7**) and Persenone A (**6**)) [16,28,56,57]. Moreover, as summarized in Supplementary Table S1, existing scientific literature obtained opposite results, and some authors report health-benefits at higher doses than doses reported to produce toxic effects, even with the application of interspecies data correction factors [29].

In prior publications, two similar studies were conducted using lactating mice models treated with single oral doses of Persin (**7**) (60–100 mg·kg^−1^ bw, purified from avocado leaves) and the authors observed necrosis of the secretory mammary gland (2 to 5 days after exposure) [28,56]; at doses greater than 100 mg·kg^−1^, other tissues were affected (e.g., myocardium) [28]. The same research group also treated lactating goats with avocado leaves from the Guatemalan race (as a single dose or as 3 doses per day) and also reported necrosis of the secretory mammary glands [58], results that were attributed to the presence of Persin (**7**) in the leaves [28]. Interestingly, the same authors confirmed the absence of detrimental effects after the administration of comparable doses of avocado leaves from the Mexican horticultural race [58]. Persin (**7**) concentrations were not measured in the study that treated lactating goats and observed negative effects [58], therefore we tried to estimate hypothetical concentrations of Persin (**7**) administered in those experiments based on the quantification of Persin (**7**) in avocado leaves conducted by Carman and Handley (1990) [3] and concentrations from our own results (Figure 4). Also, to compare prior data with our observations we applied interspecies conversion factors [29,59] for the different animal species used in prior works in order to make them equivalent to the present studies with rats (Table S1). Based on the previously reported Persin (**7**) concentrations in avocado leaves [3], we estimated that Persin (**7**) levels contained in the Guatemalan avocado leaves ingested by lactating goats [58] ranged between 38 to 95 mg·kg^−1^ bw. Even though there are some discrepancies on the horticultural race of the ’Hass’ avocado cultivar used in the present work (classified as Guatemalan [3] or hybrid [60]), based on the Persin (**7**) levels measured in the present study (Figure 4), the hypothetical exposure of lactating goats [58] to Persin (**7**) was 55 mg·kg^−1^ bw. Consistently, the presumed Persin (**7**) exposure in the goat study (38–95 or 55 mg of Persin (**7**) kg^−1^ bw) resulted considerably higher values (3–14 times) than the dose that caused necrosis of mammary gland in mouse [28,56], which is equivalent to 6.5–11 mg of Persin (**7**) kg^−1^ bw in goat (average bw of 17 kg [58]), considering interspecies dose conversion factors [29,59]. Moreover, Carman and Handley (1990) [3] reported that the Persin (**7**) level in leaves of the Mexican varieties were up to 5-times lower than the present in Guatemalan varieties, which may partially explain the absence of detrimental effects observed when avocado leaves from the Mexican varieties were tested in the lactating goat model [58].

Differing from the above-mentioned reports, Kawagishi and others (2001) [16] described the protective effects of purified avocado acetogenins, including Persin (**7**) and Persenone A (**6**), on d-galactosamine-induced liver injury. In their studies, the purified compounds were directly administered into rats’ stomachs using a catheter (as a single dose of 100 mg·kg^−1^ of bw), and then 4 h later, d-galactosamine was injected intraperitoneally. Activities of plasma alanine aminotransferase (ALT) and aspartate aminotransferase (AST) were measured after 22 h. Apparently, all the compounds exhibited strong liver injury suppressing activities, as reduced plasma ALT and AST activities were observed [16]. Another study reported a potentially beneficial bioactivity when purified Persenone A (**6**) (at dose of 25 mg·kg^−1^ bw) was administered intraperitoneally to mice, resulting in higher blood clotting times and antithrombotic activities after 24 h of exposure [57].

As discussed, prior literature has reported contradictory results regarding the safety of acetogenins in vivo. In summary, toxic effects were observed after the administration of purified acetogenins, particularly Persin (**7**) at 60–100 mg·kg^−1^ bw, in mice [28,56], while others reported health promoting bioactivities at doses of 100 mg·kg^−1^ bw in rat for Persin (**7**) and Persenone A (**6**) [16,57,61]. After the application of species-specific conversion factors [59], we observed that doses reported to produce detrimental effects in rats (30–60 mg·kg^−1^ bw) were 1.6–3-times lower that doses at which therapeutic effects were reported, without signs of toxicity. Data from our acute oral toxicity experiments (Table 3, Table 4, Table 5 and Table 6 and Figure 6) estimated the LD_50_ value in rat for Avosafe^®^ to be > 2000 mg·kg^−1^ bw. In a more precise estimation, based on the acetogenin content (94.74% *w*/*w*) of Avosafe^®^ (Figure 4), the observed LD_50_ value corresponded to 1895 mg of total acetogenins kg^−1^ bw, of which 262 and 402 mg·kg^−1^ bw was observed for Persin (**7**) and Persenone A (**6**), respectively. In the present work Persin (**7**) was not tested in a pure form, however it was present in Avosafe^®^ at concentrations that were 4–8.7-times higher than the levels previously reported to be harmful [28,56]. Acetogenins are being studied as potential substitutes for sodium nitrite (CAS No. 7632-00-0), a food additive widely used to control the germination of bacterial endospores in processed foods. LD_50_ in rats reported in literature for sodium nitrite was 77–130 mg·kg^−1^ bw [62], which was lower (15–25 times), and therefore possibly less safe than the avocado seed acetogenin-enriched extract evaluated herein. 

The current work presented various strengths in its experimental design. One advantage being that a high purity food-grade avocado seed extract of a known chemical profile (94.74% acetogenins) was studied, which was characterized and quantified using improvements in analytical and detection methodologies described in the present work. Other strengths also being the use of standardized procedures for the AMES test and for the acute oral toxicity to the rat assays (LD_50_ determination), which have been reviewed by international regulations and guidelines established by the Organization for Economic Cooperation and Development (OECD), the EC Commission, and government agencies in the United States (EPA and FDA) for the testing of chemicals [26,63,64,65]. Additional tests are recommended to further characterize the safety of acetogenin-enriched extracts as potential food additives, such as the administration of a repeated dose (sub-chronic or chronic toxicity), and reproductive and developmental or carcinogenicity studies with rodents [66].

## 4. Materials and Methods

### 4.1. Materials

Reagent grade solvents (dichloromethane and distilled water, dH_2_O) were acquired from DEQ (San Nicolas de los Garza, NL, Mexico). HPLC grade solvents (methanol, isopropanol, water) were purchased from Fisher Scientific (Springfield, NJ, USA). Analytic standards of acetogenins were purified from avocado seed in our laboratory (>97 wt% purity), as described by Rodríguez-Sánchez and others (2013b). Compounds: AcO-avocadyne (**0**), 1-acetoxy-2,4-dihydroxy-heptadec-16-yne; AcO-avocadenyne **(1)**, 1-acetoxy-2,4-dihydroxy-heptadec-12-en-16-yne; AcO-avocadene (**2**), (2*S*,4*S*)-1-acetoxy-2,4-dihydroxy-n-heptadeca-16-ene; AcO-avocadiene B (**3**), 1-acetoxy-2,4-dihydroxy-heptadeca-12,16-diene; Persenone C (**5**), (2*R*,5*E*,16*E*)-1-acetoxy-2-hydroxy-4-oxononadeca-5,16-diene; Persenone A (**6**), (2*R*,5*E*,12*Z*,15*Z*)-1-acetoxy-2-hydroxy-4-oxoheneicosa-5,12,15-triene; Persin (**7**), 2*R*,12*Z*,15*Z*)-1-acetoxy-2-hydroxy-4-oxoheneicosa-12,15-diene; and Persenone B **(8)**, (5*E*)-1-acetoxy-2-hydroxy-4-oxononadeca-5-ene. Numbers **0**–**8** next to compounds chemical names were assigned based on their chromatographic elution order (Figure 1), and additional structural information can be found in Table 1**.** Avosafe^®^, a food-grade avocado seed extract enriched in acetogenins, was kindly donated by ITESM, Centro de Biotecnologia-FEMSA (Monterrey, NL, Mexico). Food-grade propylene glycol (PG) and lysozyme were purchased from Sigma Aldrich (St. Louis, MO, USA).

### 4.2. Analyses of Acetogenin Composition

#### 4.2.1. Acetogenin Extraction for HPLC-PDA/ELSD Analysis

Avocado (*Persea americana* Mill ‘Hass’ cultivar) in a stage of commercial ripeness of the State of Michoacán, Mexico, was used and pulp and seed were manually separated. Both mature and young ‘Hass’ avocado leaves were collected from mature avocado trees growing at the Tecnologico de Monterrey Plant Nursery, Monterrey, NL, México. Three-stage Acetogenin extraction from avocado seed, pulp, and leaf was carried out as described by Rodríguez-López and collaborators (2015) [2]. Organic phases of extraction were combined and dried under nitrogen, re-suspended in HPLC-grade isopropanol (1 mg·mL^−1^), and filtered using a 0.45 mm PTFE filter for HPLC injection. Whereas Avosafe^®^, already an acetogenin-enriched extract, was directly dissolved in HPLC-grade isopropanol and adjusted at a concentration of 1 mg·mL^−1^, as previously described.

#### 4.2.2. Acetogenin Identification and Quantification by HPLC-PDA/ELSD

Acetogenin analysis was performed as reported by Salinas-Salazar and others (2015) [6], with some modifications. A 1260 Infinity series Agilent HPLC system (Santa Clara, CA, USA) was used, coupled to a G4212B photodiode array detector (PDA) G4218A and to an evaporative light scattering detector (ELSD). The PDA detector was set at 220 and 208 nm, since the latter wavelength enabled Persin (compound 7 in Table 1) identification [2]. ELSD parameters were set as follows: N_2_ pressure: 3.3 bar; temperature: 40 °C; gain: 8. The mobile phases consisted of water 100% (A) and methanol 100% (B). Solvents were pumped at 1 mL·min^−1^ using gradients of: 0−15 min, 80−95% B; 15−25 min, 95−100% B; and 25−30 min, 100–80% B, followed by 10 min re-equilibration. The column used was a Synergy Hydro RP C18 (4.6 × 250 mm, 4 µm, Phenomenex, CA, USA) kept at 35 °C. The following detector signals were considered for each compound: ELSD for compound 0, PDA at 220 nm for compounds **1** to **6**, PDA at 208 nm for compounds 7 and 8, while the rest of putative acetogenins (Table S2) were quantified as Persenone (A) equivalents, at 220 nm. Linear and second order polynomial equations were fitted to calibration curves of PDA and ELSD detectors, respectively.

#### 4.2.3. Elucidation of Chemical identity of Acetogenin Chromatographic Peaks

Elucidation of the chemical identity of compounds **1**, **2**, and **4** to **8** in Table 1 was possible by comparing their retention times and spectroscopic data to that of analytical data [4], as determined by HPLC-MS analysis. For that purpose, a 1100 Infinity series Agilent HPLC system (Santa Clara, CA, USA) coupled to an G1969A orthogonal-axis time-of-flight-mass spectrometry detector with electrospray ionization interface (ESI-TOF-MS) was used in positive-ion mode. The chromatographic method and column were the same as described above, but with 0.1% formic acid added to the mobile phases. Chemical identity of compounds **0** and **3** in Table 1 was assigned by comparing their spectroscopic data, generated by LC-ESI-MS and ESI-MS/MS, with data reported by Ramos-Jerz (2007) [14]. A Quattro Premier XE Micromass (Waters, Milford, MA, USA) triple quadrupole mass spectrometer (QQQ-MS) connected to an Acquity UPLC chromatograph (Waters, Milford, MA, USA) was used to perform LC-ESI-MS (using a chromatographic column) and ESI-MS/MS (directly infusing into the mass detector). The chromatographic separation was achieved on a BEH C18 ACQUITY column (2.1 mm × 100 mm, 1.7 µm, Waters, Milford, MA, USA), thermostatized at 35 °C. Solvent was pumped at 0.25 mL·min^−1^ using gradient of: 0–5 min, 80–84% B linear; 5–10 min, 84–88% B linear; 10–10.5 min, 88–100% B linear; 10.5–12 min, 100% B isocratic, followed by 5 min of re-equilibration. The measurements were performed in the positive ion mode (ESI^+^) controlled by the MassLynx™ software (version 4.1, Waters, Milford, MA, USA). Parameters of chromatogram and spectra acquisition (ESI-MS) included capillary, sample cone, and extraction cone voltages of 2.5 kV, 40 V, and 3 V, respectively; desolvation temperature of 40 °C; source temperature of 120 °C; cone gas (N_2_) flow of 50 L·h^−1^; desolvation gas (N_2_) flow of 250 L·h^−1^; and scan range of *m*/*z* 200–800. For ESI-MS/MS (positive ion mode), the sample was introduced into a Quattro Premier XE Micromass (Waters, Milford, MA, USA) triple quadrupole mass spectrometer via a syringe pump at a flow rate of 20 µL·min^−1^; drying gas (N_2_) flow rate was set at 200 L·h^−1^ at 40 °C; capillary, cone and extractor voltages were 4 kV, 4 V, and 5 V, respectively; and source temperature was set at 120 °C. The collision energy was set to 10 or 20 V, a spread of ±1 V, and collision gas (Ar) at 7.46 × 10^−4^ mbar with a flow rate of 0.35 mL·min^−1^.

### 4.3. Bacterial Reverse Mutation Test (AMES Test)

Avosafe^®^ mutagenicity was assessed by in vitro Bacterial Reverse Mutation test (AMES test). The evaluation was conducted under contract by Envigo Research Limited (Huntingdon, UK) in full compliance with international regulations and guidelines established by Organization for Economic Co-operation and Development (OECD), EC Commission, and U.S. organizations (EPA and FDA) the for testing of chemicals [26,63,64,65]. Four histidine-dependent auxotrophic mutant strains of *Salmonella typhimurium* (TA1535, TA1537, TA98, and TA100) and a tryptophan-dependent mutant strain of *Escherichia coli* (WP2 uvrA, pKM101) were tested in triplicate at the seven doses (5000, 1500, 500, 150, 50, 15, and 5 µg·plate^−1^) of Avosafe^®^. The highest concentration of Avosafe^®^ (5000 µg·plate^−1^) tested in this study was the standard limit concentration recommended in the regulatory guidelines for this assay [26]. Ethanol was used as vehicle control. Two independent mutation tests were performed, both in the presence and absence of S9 activator (S9 fraction (post-mitochondrial fraction supplemented with a cofactor, prepared from the liver of male Sprague Dawley rat treated with phenobarbital/5,6-benzoflavone as an enzyme inducer agent; 10% *v*/*v* for test 1 and 20% *v*/*v* for test 2), MgCl_2_ (8 mM), KCl (33 mM), sodium phosphate buffer pH 7.4 (100 mM), glucose-6-phosphate (5 mM), NADPH (4 mM), and NADH (4 mM) in water). Positive controls with and without S9 activator (2-aminoanthracene and benzo[a]pyrene; and 2-nitrofluorene, sodium azide, 9-aminoacridine and 4-nitroquinolene-1-oxide, respectively, at concentrations and for strains specified in Table 2) and negative controls with and without S9 activator (ethanol and phosphate buffer, respectively) were included in the evaluation. This was done to ensure the test system was functioning properly (positive controls) and to obtain baseline revertant frequencies for the various strains of bacteria used in the study (negative controls). All plates were counted after 69 h of incubation at 37° C. After this period, the appearance of the background bacterial lawn was examined and revertant colonies were counted using an automated colony counter (Sorcerer, Perceptive Instruments Ltd., Bury St. Edmunds, UK). Mutagenic activity of the test substance was considered positive in the case of increased concentration over the range tested and a reproducible increase in at least one or more concentrations in a number of revertant colonies per plate in at least one strain (with or without metabolic activation system). The test substance was considered to be toxic if there was a decrease in the number of revertants or thinning or absence of the background lawn.

### 4.4. Study I-Acute Oral Toxicity in Rats (Fixed Dose Method): Acute Median Lethal Oral Dose (LD_50_) and Macroscopic Pathology Analyses

#### 4.4.1. Animal Use

An acute oral toxicity test in rats was performed under contract by Envigo Research Limited (Huntingdon, UK; study number: RZE003), with the care and use of experimental animals being in compliance with the United Kingdom Animals (Scientific Procedures) Act 1986 Amendment Regulations 2012 (the Act) [67]. Healthy nulliparous and non-pregnant female RccHan^®^: WIST albino rats weighting from 155 to 186 g and eight to twelve weeks old were chosen for the experiment. They were housed alone for sighting investigations and in groups of (up to) four rats of the same sex for the main study. The animals were allowed free access to a standard rodent diet (Harlan Teklad 2014C Diet), except for overnight prior to and approximately four hours after dosing. This diet contained no added antibiotic or other chemotherapeutic or prophylactic agents. Potable water taken from the public supply was freely available via polycarbonate bottles fitted with sipper tubes. The animals were allowed to acclimatize to the conditions described below for at least 5 days before treatment. The acute oral toxicity study was carried out according to guidelines established by the OECD (No. 420) [30], Japan [68], and the EPA [69]. The appropriate dose volume of the test substance was administered to each rat by oral gavage using a plastic syringe and plastic catheter.

#### 4.4.2. Sighting Study and Main Study

In the sighting study, two fasted female rats were given a single oral gavage dose of Avosafe^®^, formulated in PG (at 30 or 200 mg of Avosafe^®^ mL^−1^) for administration at a volume of 10 mL·kg^−1^ bw, corresponding to dose level of 300 or 2000 mg·kg^−1^ bodyweight (bw), respectively. For the main study, based on the results of the sighting investigations, 4 new animals were administered 2000 mg·kg^−1^ bw.

#### 4.4.3. Observations and Macroscopic Pathology Analysis

All animals were observed for 14 days after dosing, recording clinical condition and bw. All animals were euthanized on day 15 by carbon dioxide asphyxiation. All animals were subjected to a macroscopic examination, which consisted of opening the cranial, thoracic, and abdominal cavities. The macroscopic appearance of the brain, caecum, duodenum, heart, kidneys, small and large intestine, liver, lungs and bronchi, spleen, stomach, subcutaneous tissue, and urinary bladder was recorded.

### 4.5. Study II-Acute Oral Toxicity in Rats (Single Dose of 2000 mg·kg^−1^): Hematology, Serum Biochemistry, and Histological Examination

#### 4.5.1. Animal Use and Treatment

The study was performed at Tecnologico de Monterrey (Monterrey, N.L., México) after approval of the Institutional Committee for the Right Use and Care of Laboratory Animals (ethical approval code: 2014-009). Procedures were conducted following guidelines established in the Mexican National Protection Laws on Animal Protection and the General Health Law Related to Health Research (NOM-062-Z00-1999). Nulliparous and non-pregnant female RccHan^®^: WIST albino rats weighing 200–250 g were obtained from Scientific Services Tetrarium. Animals were distributed in two groups (3 animals per group) based on their bw using a balanced design. Animals were housed in groups at constant temperature (21–24 °C) and light–dark cycles of 12 h and allowed to acclimatize for 5 days before treatment. Food and water were provided ad libitum, except for overnight prior to and approximately four hours after dosing, as well as overnight before scheduled necropsy. Avosafe^®^ formulated in PG (vehicle) was administered to each rat (2000 mg of Avosafe^®^ kg^−1^ bw) by oral gavage using a plastic syringe and plastic catheter. Control animals only received the vehicle (2000 mg of PG kg^−1^ bw). All animals were observed throughout the experimental period (14 days after dosing). 

#### 4.5.2. Clinical Observations and Body Weight

The clinical conditions of treated animals were monitored over 14 days after dosing, at hourly intervals during the first 6 h of day 1 and daily on subsequent days. Recorded clinical condition included changes in skin and fur appearance, behavioral patterns (posture, movement, sleep), urine, and fluid secretion and excretion. The body weight (bw) of each animal was recorded prior to dosing (day 1) and subsequently on day 8 and 15, and bw changes from days 1–8 and from days 8–15 were calculated for each animal group.

#### 4.5.3. Hematology and Serum Biochemistry Analysis

On day 14, overnight fasted animals were anesthetized with sodium pentobarbital before blood was collected from the tail vein into tubes containing either EDTA (for hematology parameters) or lithium heparin (for clinical chemistry parameters). Hematologic analysis was carried out (red blood cell count, hemoglobin, hematocrit, mean corpuscular volume, mean corpuscular hemoglobin, mean corpuscular hemoglobin concentration, platelet count, white blood cell count, and white blood cell differential based on percentage, including neutrophils, lymphocytes, monocytes, eosinophils, and basophils). For biochemical analysis, we determined levels of aspartate aminotransferase, alanine aminotransferase, alkaline phosphatase, creatine phosphokinase, total bilirubin, glucose, total cholesterol, triglycerides, total protein, albumin, albumin/globulin ratio, blood urea nitrogen, creatinine, inorganic phosphorus, calcium ions, sodium ions, potassium ions, and chloride ions. For both studies, an automated CELL-DYN Ruby Hematological Analyzer (Illinois, USA) was employed to carry the blood samples analysis.

#### 4.5.4. Necropsy and Histopathology

Animals were euthanized on day 14 by an overdose of sodium pentobarbital. The macroscopic appearance and weight of heart, brain, liver, and pair of kidneys were recorded. Ratios of organ weight relative to body weight were calculated (grams per 100 g of bw). Organs were fixed in formalin, dehydrated, and embedded in paraffin for further staining with hematoxylin and eosin pathology score. Organs for which there were no abnormalities in macroscopic appearance or weight relative to body weight were retained in fixative and not analyzed histopathologically.

### 4.6. Statistical Analysis

Results were expressed as means ± standard deviation. Statistically significant differences among groups were analyzed employing one-way analysis of variance (ANOVA). Differences were considered significant at a level of *p* < 0.05. Grouping by Tukey’s Honestly Significant Difference (HSD) was performed (α < 0.05). Differences between the vehicle control and the treated group in in vivo experiments were estimated by LSMean Dunnett’s test (*p* < 0.05). Statistical analysis was performed using JMP^®^ software version 13.0.0 (SAS Institute Inc., Cary, NC, USA).

## 5. Conclusions

A multiple detection strategy that used HPLC-PDA/ELSD, HPLC-ESI-TOF-MS, and ESI-MS/MS was developed for identification and quantification of additional acetogenins present in avocado tissues (pulp, seed, and leaves) and in an enriched food-grade extract obtained from avocado seed. The use of low-energy CID aided the assignment of the location of unsaturations in acetogenins, resulting in the confirmation of the chemical structures of two molecules (AcO-avocadyne (0) and AcO-avocadiene B (3)), as present in Avosafe^®^, and together with other seven major peaks quantified 94.74 ± 5.77% *w*/*w* of its total solids. Eleven other small chromatographic peaks exhibiting the characteristic ion pattern of acetogenins were also identified as present in the enriched extract and individually contributed concentrations that ranged between 0.34 to 1.41% to the Avosafe^®^ total solids (quantified as Persenone A equivalents).

Quantification of individual acetogenins found in avocado tissues indicated that seed contained the highest total concentrations (2.31 ± 0.29% dw). Seed profiles and concentrations of individual acetogenins were different when compared with those of pulp and leaves, the latter featuring the highest Persin (**7**) concentrations (contributed 52% to the total acetogenins). Safety evaluations indicated that Avosafe^®^ was non-mutagenic, had an acute median lethal oral dose (LD_50_) for rats estimated to be higher than the maximum concentration tested (>2000 mg·kg^−1^), and showed no signs of macroscopic abnormalities in organs. Mean body weight, hematological, and biochemical parameters were also normal after 14 days of administration of the single oral dose of 2000 mg·kg^−1^. More tests are desirable to further characterize the safety of acetogenins as food additives; however, the present work advanced scientific knowledge on this family of lipid derivatives contained in the widely consumed avocado fruit.

## 6. Patents

Some of the potential commercial uses of acetogenins in the food, pharmaceutical, and personal care industries are protected under patent applications WO/2012/042404 and US 20180103671A1.

## Figures and Tables

**Table 1:** Chemical structure of acetogenins detected as present in avocado fruit (*Persea americana*).

Compound (Number) ^a^	Elution Time (min) ^b^/Detector	[M + H]^+^/Ions Pattern (*m*/*z*) ^c^	Molecular Formula	Structure	References
AcO ^d^-avocadyne (**0**)	9.19/ELSD	327/349, 309, 267, 249, 231	C_19_H_34_O_4_	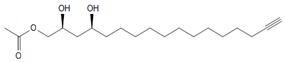	[14]
AcO-avocadenyne (**1**)	11.61/PDA @ 220 nm	325/347, 307, 265	C_19_H_32_O_4_	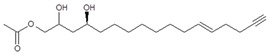	[2,14]
AcO-avocadene (**2**)	13.48/PDA @ 220 nm	329/351, 311, 269, 251	C_19_H_36_O_4_	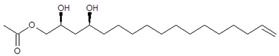	[15]
AcO-avocadiene B (**3**)	13.92/PDA @ 220 nm	327/349, 309, 267, 249, 231	C_19_H_34_O_4_	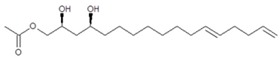	[2,14]
Persediene (**4**)	15.11/PDA @ 220 nm	353/375, 335, 293	C_21_H_36_O_4_	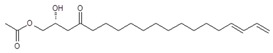	[4]
Persenone C (**5**)	15.40/PDA @ 220 nm	353/375, 335, 293	C_21_H_36_O_4_	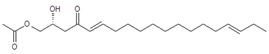	[4]
Persenone A (**6**)	16.14/PDA @ 220 nm	379/401, 361, 319, 301	C_23_H_38_O_4_	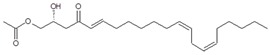	[15]
Persin (**7**)	17.50/PDA @ 208 nm	381/403, 363, 321, 303	C_23_H_40_O_4_	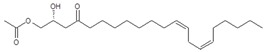	[16]
Persenone B (**8**)	17.63/PDA @ 208 nm	355/377, 337, 295	C_21_H_38_O_4_	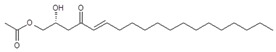	[17]

^a^ Compound numbers from **0** to **8** were assigned according to their elution order from the HPLC column, as shown in Figure 1. ^b^ Elution times of chromatographic peaks from HPLC column, as shown in Figure 1A. ^c^ MS/TOF detection using electrospray ionization interface in positive-ion mode of analysis. ^d^ AcO: 1-acetate.

**Table 2:** Fold increase in revertant colony numbers of tester strains (relative to their vehicle) following the exposure to positive controls and to an avocado seed extract enriched in acetogenins (Avosafe^®^), with and without metabolic activation.

	Test Substance ^a^	Concentration (µg·Plate^−1^)	Increase in Revertant Bacterial Colony Numbers				
			*S. typhimurium*				*E. coli*
			TA98	TA100	TA1535	TA1537	WP2 uvrA
Without metabolic activation	**Avosafe^®^**	5000.0	1.0	0.0	0.5	0.0	0.4
	**2NF**	2.0	3.9	-	-	-	-
	**NaN3**	2.0	-	7.2	-	-	-
	**NaN3**	2.0	-	-	27.6	-	-
	**AAC**	50.0	-	-	-	21.5	-
	**NQO**	2.0	-	-	-	-	14.9
With metabolic activation	**Avosafe^®^**	5000.0	0.5	0.1	0.3	0.0	0.5
	**B[a]P**	5.0	4.1	-	-	-	-
	**AAN**	5.0	-	12.3	-	-	-
	**AAN**	5.0	-	-	18.3	-	-
	**B[a]P**	5.0	-	-	-	5.6	-
	**AAN**	10.0	-	-	-	-	5.4

Note: ^a^ 2NF = 2-Nitrofluorene; NaN3 = Sodium azide; AAC = 9-Aminoacridine; NQO = 4-Nitroquinoline-1-oxide; B[a]P = Benzo[a]pyrene; AAN = 2-Aminoanthracene.

**Table 3:** Clinical observation of rats after acute oral administration (2000 mg·kg^−1^) of a food-grade extract from avocado seed (Avosafe^®^) with an acetogenin purity of 94.74%.

Observation	Dosage Groups ^a^			
	0 mg·kg^−1^		2000 mg·kg^−1^	
	6 h	14 Day	6 h	14 Day
Fur appearance	Normal	Normal	Normal	Normal
Posture	Normal	Normal	Normal	Normal
Movement	Diminished	Normal	Diminished	Normal
Sleep	Normal	Normal	Normal	Normal
Diarrhea	Normal	Normal	Normal ^b^	Normal

^a^ Number of animals per group = 3. ^b^ A single soft evacuation was observed in one animal during the first 6 h after administration.

**Table 4:** Hematologic values for female rats after 14 days of being exposed to a single dose (2000 mg·kg^−1^) of a food-grade extract from avocado seed (Avosafe^®^), with an acetogenin purity of 94.74%.

Parameter (Units)	Dosage Group ^a^	
	0 mg·kg^−1^	2000 mg·kg^−1^
Red blood cell count (×10^6^ μL^−1^)	6.5 ± 1.4	7.2 ± 0.1
Hemoglobin (g·dL^−1^)	14.2 ± 0.7	12.8 ± 0.4 *
Mean corpuscular hemoglobin (pg)	22.8 ± 6.6	17.7 ± 0.5
Mean corpuscular hemoglobin concentration (%)	32.9 ± 0.5	32.0 ± 0.4
Platelet count (×10^3^ μL^−1^)	666.0 ± 381.1	847.7 ± 126.5
White blood cell count (×10^3^ μL^−1^)	3.7 ± 1.8	3.9 ± 1.0
Neutrophils (%)	25.8 ± 13.3	27.1 ± 4.8
Lymphocytes (%)	67.8 ± 12.5	66.7 ± 6.2
Monocytes (%)	0.9 ± 0.3	0.3 ± 0.1
Eosinophils (%)	2.0 ± 0.4	4.2 ± 1.4
Basophils (%)	3.5 ± 1.2	1.7 ± 0.2

^a^ Values represent mean ± SD (*n* = 3). * Significantly different from vehicle control, according to Dunnett’s test (*p* < 0.05).

**Table 5:** Serum biochemical parameters in female rats, after 14 days of being exposed to a single dose (2000 mg·kg^−1^) of a food-grade extract from avocado seed (Avosafe^®^), with an acetogenin purity of 94.74%.

Parameter (Units)	Dosage Group ^a^	
	0 mg·kg^−1^	2000 mg·kg^−1^
Aspartate aminotransferase (U·L^−1^)	159.3 ± 14.2	120.3 ± 9.0
Alanine aminotransferase (U·L^−1^)	51.7 ± 5.5	45.0 ± 1.0
Alkaline phosphatase (U·L^−1^)	142.0 ± 37.2	134.7 ± 9.0
Total bilirubin (mg·dL^−1^)	>0.1 ± 0.0	>0.1 ± 0.0
Glucose (mg·dL^−1^)	118.0 ± 33.9	157.3 ± 19.4 *
Total cholesterol (mg·dL^−1^)	60.3 ± 4.5	63.7 ± 3.8
Triglycerides (mg·dL^−1^)	49.3 ± 19.6	57.0 ± 13.0
Total protein (g·dL^−1^)	6.6 ± 0.3	6.2 ± 0.5
Albumin (g·dL^−1^)	1.5 ± 0.2	1.5 ± 0.2
Albumin/globulin ratio	0.3 ± 0.0	0.3 ± 0.0
Blood urea nitrogen (mg·dL^−1^)	22.4 ± 4.9	20.3 ± 4.8
Creatinine (mg·dL^−1^)	0.6 ± 0.0	0.6 ± 0.1
Inorganic phosphorus (mg·dL^−1^)	8.6 ± 2.0	7.6 ± 0.4
Calcium ion (mEq·L^−1^)	10.4 ± 0.1	10.3 ± 0.3
Sodium ion (mEq·dL^−1^)	143.3 ± 1.5	141.4 ± 2.4
Potassium ion (mEq·dL^−1^)	4.6 ± 1.0	4.3 ± 0.2
Chloride ion (mEq·dL^−1^)	100.4 ± 0.6	97.6 ± 2.1

^a^ Values represent mean ± SD (*n* = 3). * Significantly different from vehicle control, according to Dunnett’s test (*p* < 0.05).

**Table 6:** Organ weights relative to body weight (g·100 g^−1^ bw) for female rats, after 14 days of being exposed to a single dose (2000 mg·kg^−1^) of a food-grade extract from avocado seed (Avosafe^®^), with an acetogenin purity of 94.74%.

Organ (% bw)	Dosage Group ^a^	
	0 mg·kg^−1^	2000 mg·kg^−1^
Brain	0.72 ± 0.09	0.76 ± 0.05
Heart	0.37 ± 0.02	0.37 ± 0.01
Liver	4.21 ± 0.43	4.87 ± 0.24
Kidney ^b^	0.80 ± 0.05	0.77 ± 0.04

^a^ Values represent mean ± SD (*n* = 3). ^b^ Weighted in pair. No significant different was found between the vehicle control and the treated group, according to Dunnett’s test (*p* < 0.05).

## Supplementary Materials

The following are available online, Figure S1: LC-ESI-MS spectra of compound 0 (A) and 3 (B), Figure S2: ESI-MS/MS spectra of AcO-avocadene (2) obtained by low-energy collision-induced dissociation (CID) of the precursor ion at *m/z* 329 and daughter ion at 251. Data shown in panels (A), (B), and (C) for collision energies of 10, 20, and 30 eV, respectively, Table S1: Summary of in vivo toxicological and health promoting effects of purified acetogenins from avocado fruit (*Persea americana*), as reported in scientific literature, Table S2: Ion pattern of minor peaks present in a food-grade acetogenin-enriched extract from avocado seed (Avosafe^®^), as determined by HPLC-ESI-TOF-MS, Table S3: Fold increase in revertant colony numbers of tester strains relative to their vehicle (ethanol) following exposure to positive controls and to a food-grade extract from avocado seed (Avosafe^®^), with and without metabolic activation, Table S4: Summary of the macroscopic findings after exposure (single oral dose) of female rats to fixed doses a food-grade extract from avocado seed (Avosafe^®^), with an acetogenin purity of 94.74%.
