# Supplementary material for: Chemical Profile and Safety Assessment of a Food-Grade Acetogenin-Enriched Antimicrobial Extract from Avocado Seed
Source: Molecules. 2019 Jun 26;24(13):2354. doi: 10.3390/molecules24132354 (PMC6651291; doi:10.3390/molecules24132354)
Supplement: Supplementary file 1 [file molecules-24-02354-s001.pdf]

Supplementary Material

Table S1. Summary of *in vivo* toxicological and health promoting effects of purified acetogenins from avocado fruit (*Persea americana*) as reported in scientific literature.

Purified acetogenin / Tissue source	Animal Model / Administration form	Observation period	Observation / Dose	Dose expressed for rat[a]	Reference
Persin (**7**[b]) / Purified from leaves	Lactating mice / Oral gavage, single dose	From 3 to 5 days after exposure to purified Persin (**7**), the dams were euthanized and mammary glands and heart were fixed, stained and subjected to pathological examination,	Necrosis of the secretory mammary gland at doses of 60-100 mg kg^{-1} of bw[c]. Myocardial tissue damage at doses > 100 mg kg^{-1} of bw.	30 mg - 50 mg kg^{-1} of bw.	[29]
Persin (**7**) / Purified from leaves	Lactating mice / Oral gavage, single dose	48 h post-dosing, the dams were euthanized and mammary glands were fixed, stained and subjected to pathological examination. Pup bw was measured daily after dosing and used as an indirect indicator of mammary gland function and milk production.	Necrosis and/or apoptosis of the mammary gland, consequential inhibition of milk flow and in pups' bw gain at doses of 100 mg kg^{-1} of bw. There were no visible signs of persin effects on other tissues examined.	50 mg kg^{-1} of bw.	[57]
Persenone A (**6**) and Persin (**7**) / Purified from pulp	Rats / Oral gavage, single dose	After 4h of the administration of each compound, liver injury was induced (by injecting D-galactosamine intraperitoneally) and 22 h later rats were euthanized to obtain blood.	Liver protection: Reduced plasma (ALT) and (AST) indicating potent liver injury suppression at doses of 100 mg kg^{-1} of bw.	100 mg kg^{-1} of bw.	[16]
Persenone A (**6**) / Purified from pulp	CD1 Mice / Intraperitoneally single dose	After 24 h of administration, thrombosis was induced by a surgical model and 1 h later the vascular segment were fixed, stained and subjected to pathological examination,	Increase in blood clotting times (2-fold) and attenuation of thrombus formation (71%) at doses of 25 mg kg^{-1} of bw.	12.6 mg kg^{-1} of bw.	[58]

[a] Considering interspecies correction factors [30].

[b] Compound numbers are in reference to chromatographic elution times as indicated in **Table 1**.

[c] bw: body weight.

Table S2. Ion pattern of minor peaks present in a food-grade acetogenin-enriched extract from avocado seed (Avosafe®), as determined by HPLC-ESI-TOF- MS.

Elution time from HPLC column (min) [a]	[M+H]+ [b] /Ions Pattern (m/z)	Possible chemical identity and structure [c]	Reference
7.91	323/<br>667,345,305	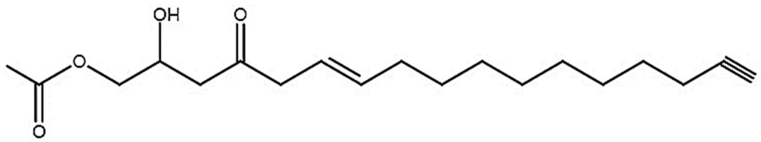<br>1-acetoxy-2-hydroxy-4-oxo-heptadec-5-en-16-yne	[14]
9.19	325/<br>671, 347	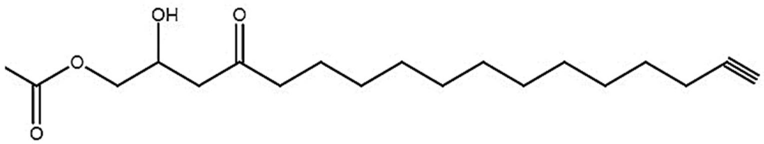<br>1-acetoxy-2-hydroxy-4-oxo-heptadec-16-yne (AcO-Avocadynone)<br>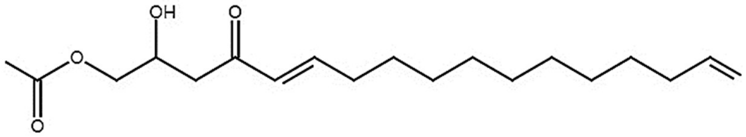<br>1-acetoxy-2-hydroxy-4-oxo-heptadec-5,16-diene	[14]

Table S2. Ion pattern of minor peaks present in a food-grade acetogenin-enriched extract from avocado seed (Avosafe®), as determined by HPLC-ESI-TOF- MS (Continuation)

Elution time from HPLC column (min) [a]	$[M+H]^{+}$ [b] /Ions Pattern (m/z)	Possible chemical identity and structure [c]	Reference
10.84	369/ 759, 391	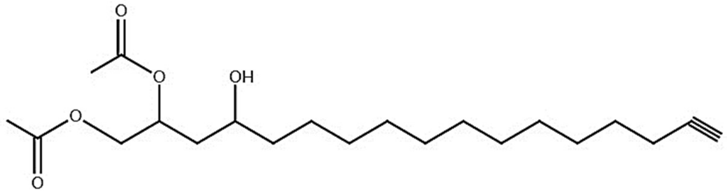 1,2-diacetoxy-4-hydroxy-n-heptadeca-16-yne	[25]
11.45	369/ 759, 391	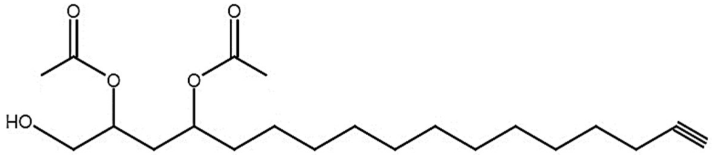 2,4-diacetoxy-1-hydroxy-n-heptadeca-16-yne	
12.21	327/ 675, 349, 301	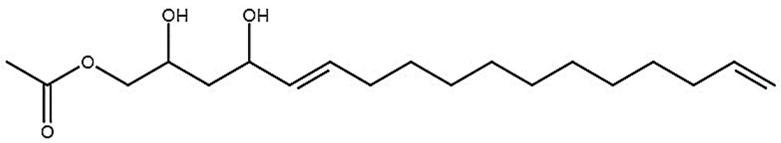 1-acetoxy-2,4-dihydroxy-heptadec-5,16-diene	[14]
14.96	337/ 695, 359, 319	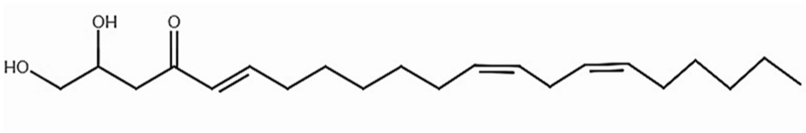 1,2-dihydroxy-4-oxo-heneicosa-5,12,15-triene	[26]

Table S2. Ion pattern of minor peaks present in a food-grade acetogenin-enriched extract from avocado seed (Avosafe®), as determined by HPLC-ESI-TOF- MS (Continuation)

Elution time from HPLC column (min) [a]	$[M+H]^{+}$ [b] /Ions Pattern (m/z)	Possible chemical identity and structure [c]	Reference
15.09	371/ 763, 393, 353, 311	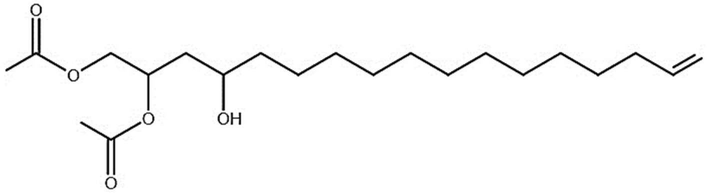 1,2-diacetoxy-4-hydroxy-n-heptadeca-16-ene	[25]
15.78	371/ 763, 393, 353, 311	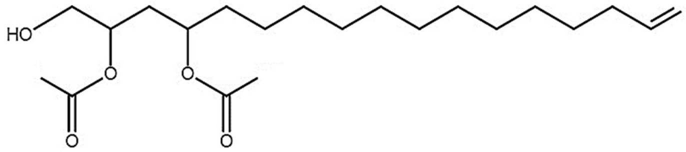 2,4-diacetoxy-1-hydroxy-n-heptadeca-16-ene	
15.34	355/ 731, 377, 337, 319, 295	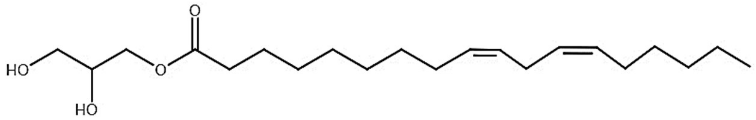 1-O-linoleoyl-glycerol (1-Monolinolein)	
17.02	355/ 731, 377, 295	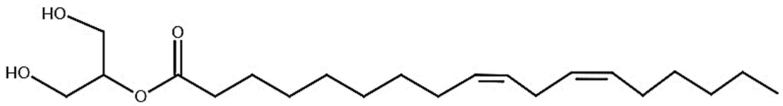 2-O-linoleoyl-glycerol (2-Monolinolein)	[26]

Table S2. Ion pattern of minor peaks present in a food-grade acetogenin-enriched extract from avocado seed (Avosafe®), as determined by HPLC-ESI-TOF- MS (Continuation)

Elution time from HPLC column (min) [a]	$[M+H]^{+}$ [b] /Ions Pattern (m/z)	Possible chemical identity and structure [c]	Reference
16.04	379/ 779, 401, 361, 319	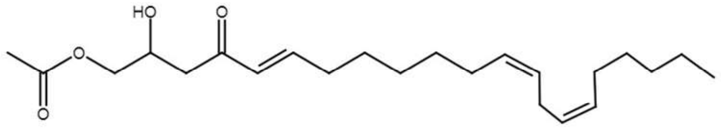 (2S,5E,12Z,15Z)-form 1-acetoxy-2-hydroxy-4-oxo-heneicosa-5,12,15-triene	[26]
18.55	381/ 403, 363, 321, 303	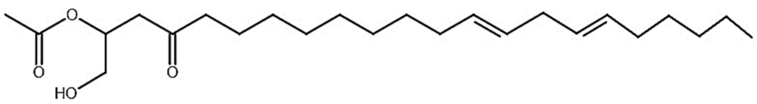 1-hydroxy-2-acetoxy-4-oxo-henicosa-12,15-diene (Isopersin)	[16]
		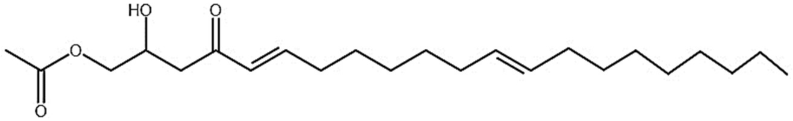 1-acetoxy-2-hydroxy-4-oxo-heneicosa-5,12-diene	[16]
		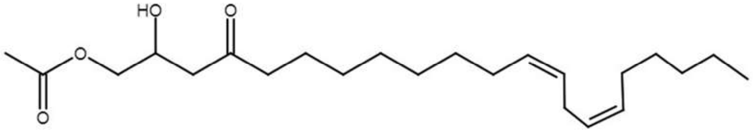 (2S,12Z,15Z)-form 1-acetoxy-2-hydroxy-4-oxo-heneicosa-12,15-diene	[26]

[a] Elution times of chromatographic peaks from HPLC column as shown in **Figure 1B.**

[b] Proposed from the observed ion pattern.

[c] Proposed based on the correspondence of observed ion pattern and previous reports on literature.

Table S3. Fold increase in revertant colony numbers of tester strains relative to their vehicle (ethanol) following exposure to positive controls and to a food-grade extract from avocado seed (Avosafe®)[a], with and without metabolic activation.

	Concentration (µg/plate)	Increase in Revertant Bacterial Colony Numbers				
		S. typhimurium				*E. coli*
		**TA98**	**TA100**	**TA1535**	**TA1537**	**WP2 uvrA**
Without metabolic activation	5.0	1.1	0.8	1.2	1.5	1.0
	15.0	1.3	0.9	1.1	1.7	0.9
	50.0	1.0	0.8	0.9	0.8	0.9
	150.0	0.9	0.8	1.1	1.1	0.9
	500.0	0.6	0.0	0.8	0.5	1.0
	1500.0	0.7	0.0	0.4	0.0	0.8
	5000.0	1.0	0.0	0.5	0.0	0.4
With metabolic activation	5.0	0.9	1.0	1.3	1.0	1.0
	15.0	0.9	0.7	1.1	0.9	1.1
	50.0	0.9	0.8	1.1	1	1.1
	150.0	1.1	0.9	0.9	0.6	1.2
	500.0	1.0	0.2	1	0.3	0.9
	1500.0	0.4	0.0	0.6	0.2	0.7
	5000.0	0.5	0.1	0.3	0.0	0.5

[a] Acetogenin content of Avosafe® was 94.74 % w/w, as determined in the present work (**Figure 4**).

Table S4. Summary of the macroscopic findings after exposure (single oral dose) of female rats to fixed doses a food-grade extract from avocado seed (Avosafe®), with an acetogenin purity of 94.74 %.

	Sighting investigations		Main study			
	300 mg kg^{-1}	2,000 mg kg^{-1}	2,000 mg kg^{-1}			
Fate of animal	K[a]	K	K	K	K	K
Day of death	15	15	15	15	15	15
**Tissues examined**			**Findings**			
Subcutaneous tissue	ND[b]	ND	ND	ND	ND	ND
Brain	ND	ND	ND	ND	ND	ND
Heart	ND	ND	ND	ND	ND	ND
Lungs	ND	ND	ND	ND	ND	ND
Liver	ND	ND	ND	ND	ND	ND
Spleen	ND	ND	ND	ND	ND	ND
Kidneys	ND	ND	ND	ND	ND	ND
Stomach	ND	ND	ND	ND	ND	ND
Duodenum	ND	ND	ND	ND	ND	ND
Small Intestines	ND	ND	ND	ND	ND	ND
Large Intestines	ND	ND	ND	ND	ND	ND
Caecum	ND	ND	ND	ND	ND	ND
Urinary Bladder	ND	ND	ND	ND	ND	ND

[a] K: Killed at study termination, [b] ND: No abnormalities detected.

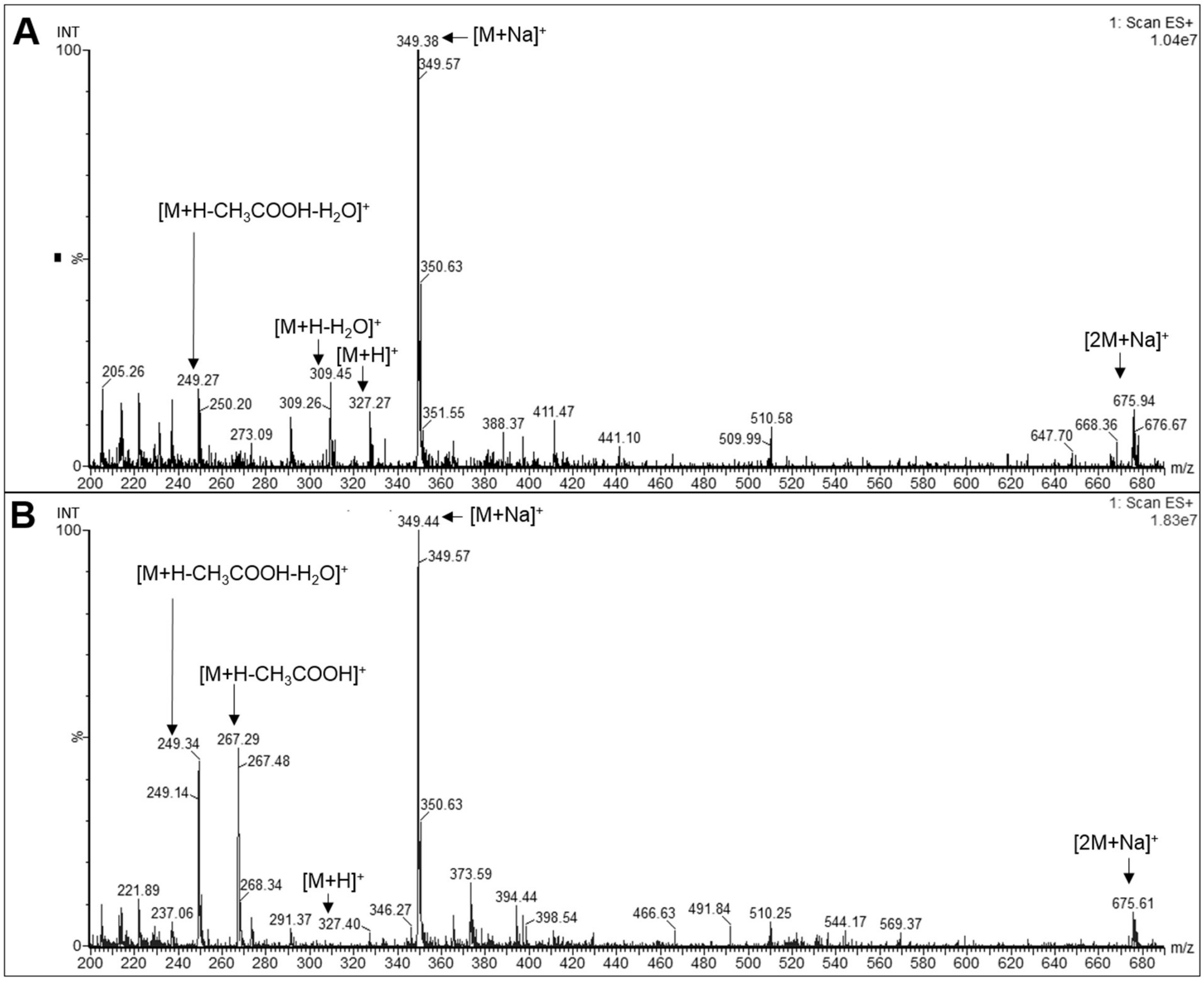

Figure S1. LC-ESI-MS spectra of compound **0** (**A**) and **3** (**B**). Details of their chemical identity are specified in **Table 1**.

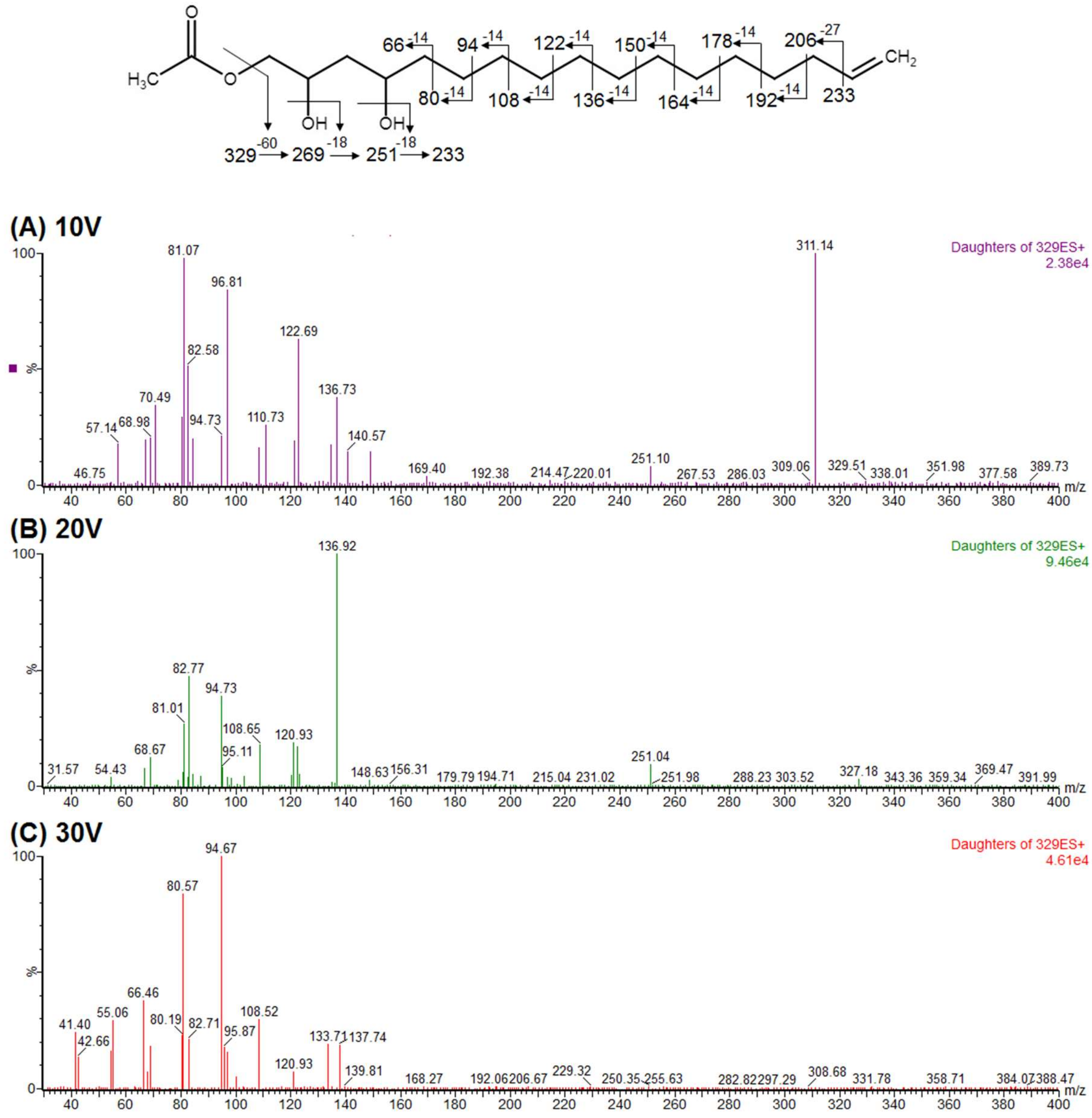

Figure S2. ESI-MS/MS spectra of AcO-avocadene (**2**) obtained by low-energy collision-induced dissociation (CID) of the precursor ion at *m*/*z* 329 and daughter ion at 251. Data shown in panels (**A**), (**B**) and (**C**) for collision energies of 10, 20 and 30 eV, respectively.
